# Supplementary material for: Building momentum: A computational account of persistence toward long-term goals
Source: PLoS Comput Biol. 2025 May 14;21(5):e1013054. doi: 10.1371/journal.pcbi.1013054 (PMC12101773; doi:10.1371/journal.pcbi.1013054)
Supplement: S1 Appendix — (PDF) [file pcbi.1013054.s001.pdf]

S1 Appendix: Experimenting with variants of the prospective model

Variants of prospection

Prospective + Differential Learning (DL) This model employs asymmetric learning rates for positive and negative prediction errors that incorporate biased learning from positive and null outcomes.

$$M_g^{(t)} = M_g^{(t-1)} + \eta_+(1 - M_g^{(t-1)}) \tag{1}$$

$$M_g^{(t)} = M_g^{(t-1)} + \eta_-(0 - M_g^{(t-1)}) \tag{2}$$

$$\tag{3}$$

Prospective + Momentum (M) This model employs the conceptualization of momentum by machine learning[1] and creates inertia in learning by considering a running average of past gradients. Without momentum:

$$M_g^{(t)} = v_t^s + \eta(I_g^{(t)} - M_g^{(t-1)}) \tag{4}$$

With momentum:

$$m_t = m_{t-1} + \eta_m(I_g^{(t)} - M_g^{(t-1)} - m_{t-1}) \tag{5}$$

$$M_g^{(t)} = M_g^{(t-1)} + \eta(m_t + I_g^{(t)} - M_g^{(t-1)}) \tag{6}$$

$$\tag{7}$$

Prospective + DL + M This variant incorporates both asymmetric learning rates and incorporates running average of past gradients.

Figure A3 shows the AIC and BIC score comparisons of different variants of prospection in experiment 2; evidently the simplest variant explains the task better than the augmented variants.

Prospective + Hyperbolic This variant incorporates hyperbolic discounting to discount future outcomes in the prospective model. Probability belief estimates are used to roll-out expectations about the trials-to-completion (delay-to-reward D) and the suit collection reward is discounted by a hyperbolic discount factor. ($\gamma = \frac{1}{1+kD}$, $k \in [0, 1]$). There is no significant difference between this variant and the original prospective variant with exponential discounting.

References

[1] Eldar, E., Rutledge, R. B., Dolan, R. J. & Niv, Y. Mood as representation of momentum. *Trends in Cognitive Sciences* **20**, 15–24 (2016).
