# Supplementary figures and images for: Building momentum: A computational account of persistence toward long-term goals
Source: PLoS Comput Biol. 2025 May 14;21(5):e1013054. doi: 10.1371/journal.pcbi.1013054 (PMC12101773; doi:10.1371/journal.pcbi.1013054)

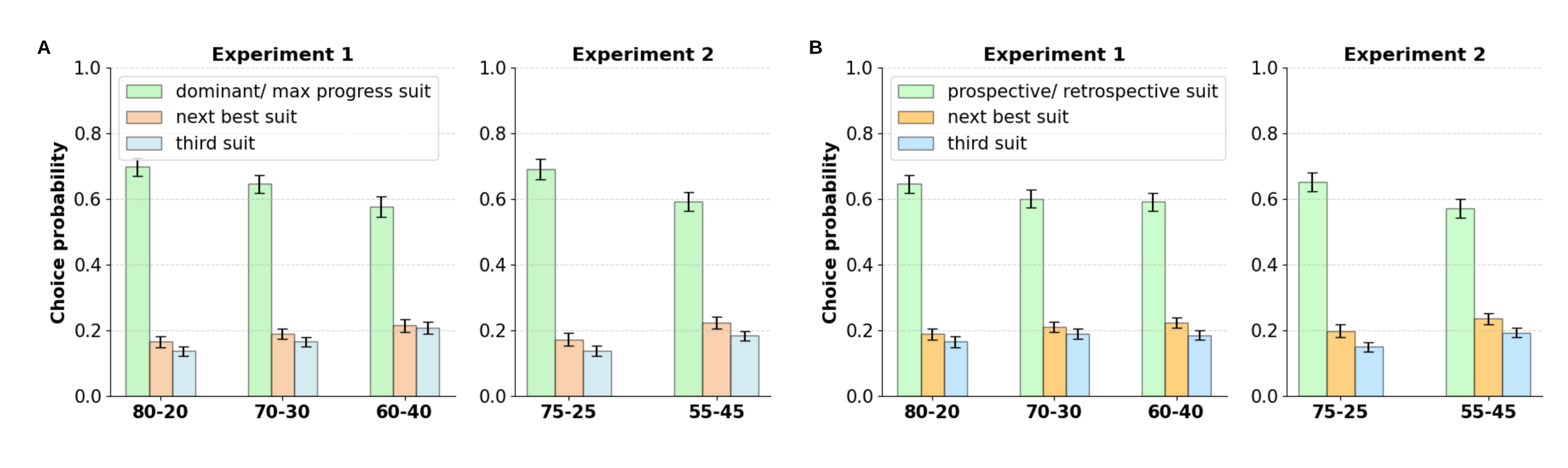

Supplement: S1 Fig — A. When the dominant and maximum progress suits are the same, participants predominantly choose the dominant/maximum progress option because it is devoid of goal conflict between accrued progress and the current rate of progress. B. Similar pattern of preference is shown when suits are classified as prospective and retrospective (based on predictions of the prospective agent optimized for the task). (TIF) [file pcbi.1013054.s002.tif]

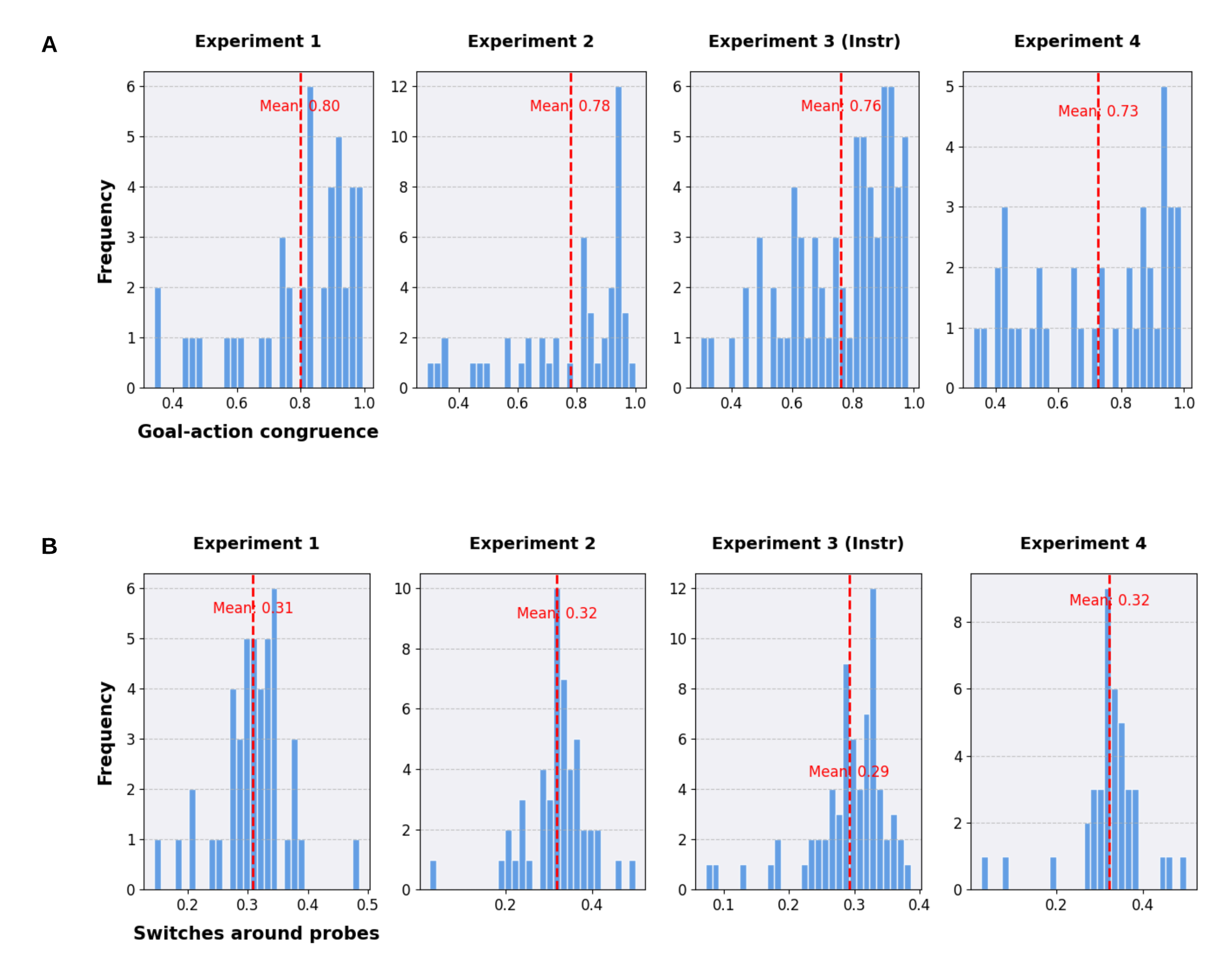

Supplement: S2 Fig — Figure shows the frequency distribution of the proportion of rounds where the participant performed action in congruence with the objective probe in the next round. A. Switches after goal probes Frequency distribution of the proportion of the total number of switches made by participants that occur in the round following the goal probe. (TIF) [file pcbi.1013054.s003.tif]

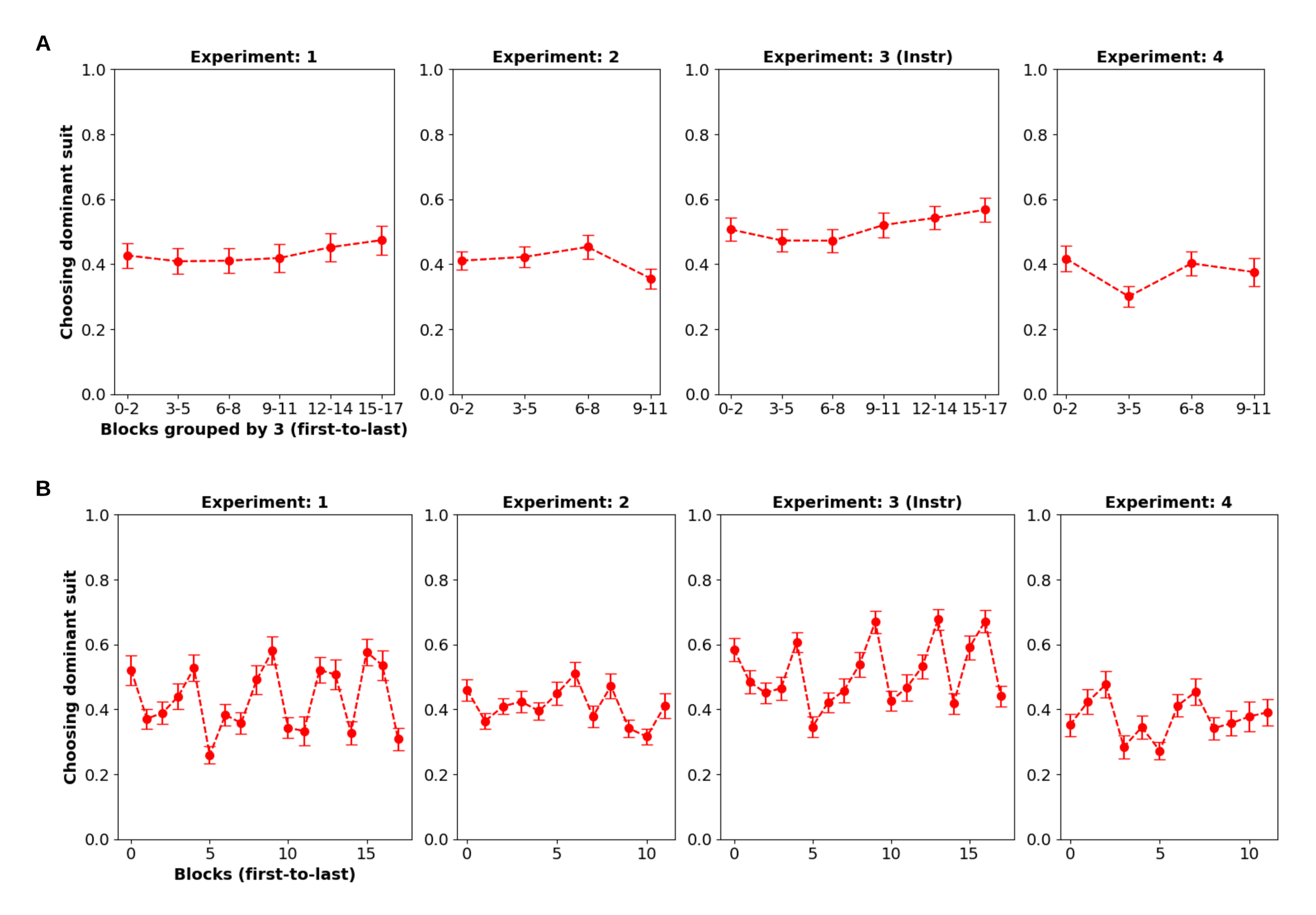

Supplement: S3 Fig — Blocks are split into groups of 3 to track progress throughout the task. There is no indication of increased persistence at the end of the task (last three blocks) compared to the beginning (first three blocks), except in experiment 2 where there is a marginal decrease in preference towards the dominant suit at the end. B. Proportion of the choice of dominant suit with no grouping of blocks. Oscillations in dominant choice proportion correspond to the block condition encountered. (TIF) [file pcbi.1013054.s004.tif]

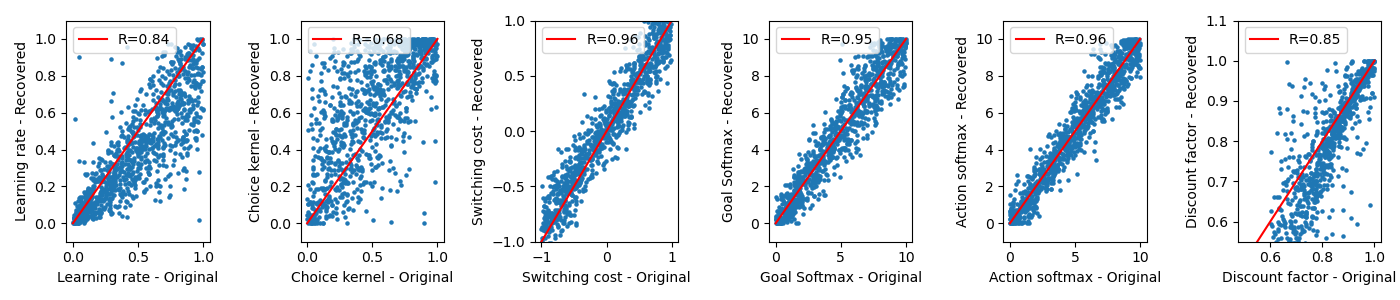

Supplement: S4 Fig — 1000 randomly generated parameter vectors were used to generate synthetic behavior and were recovered by fitting the model (recovery analysis in experiment 1). (TIF) [file pcbi.1013054.s005.tif]

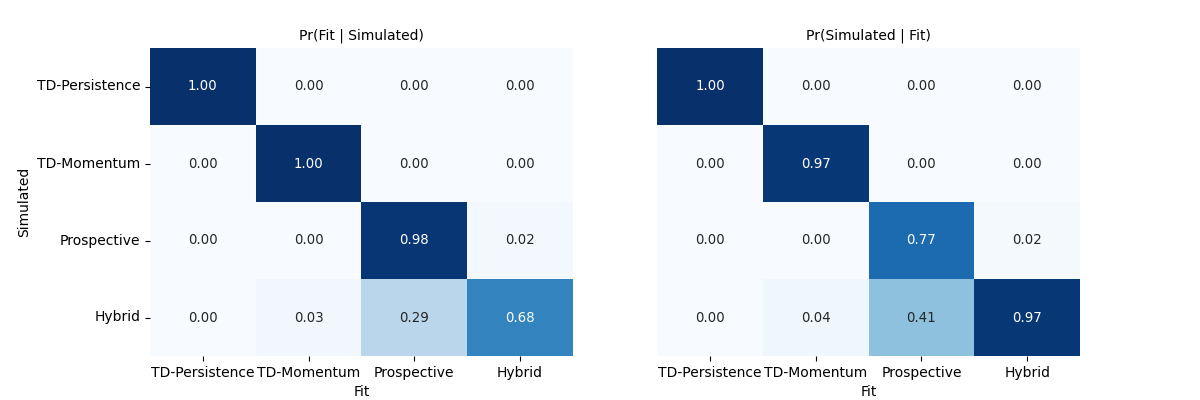

Supplement: S5 Fig — Data generating model offers the best explanation for all competing hypotheses (recovery analysis in experiment 1). (TIF) [file pcbi.1013054.s006.tif]

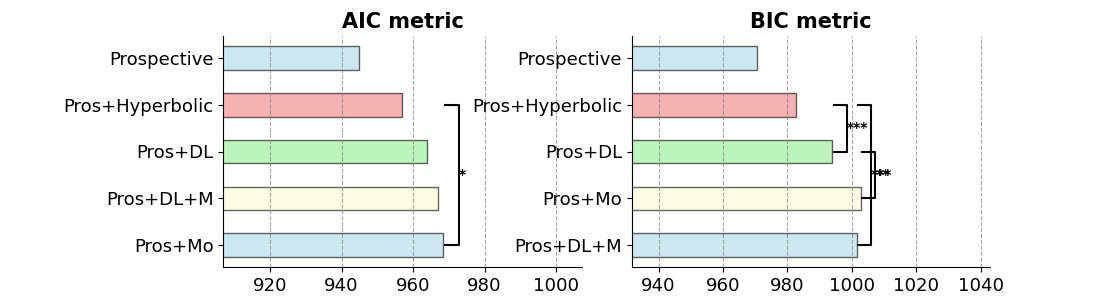

Supplement: S6 Fig — (TIF) [file pcbi.1013054.s007.tif]

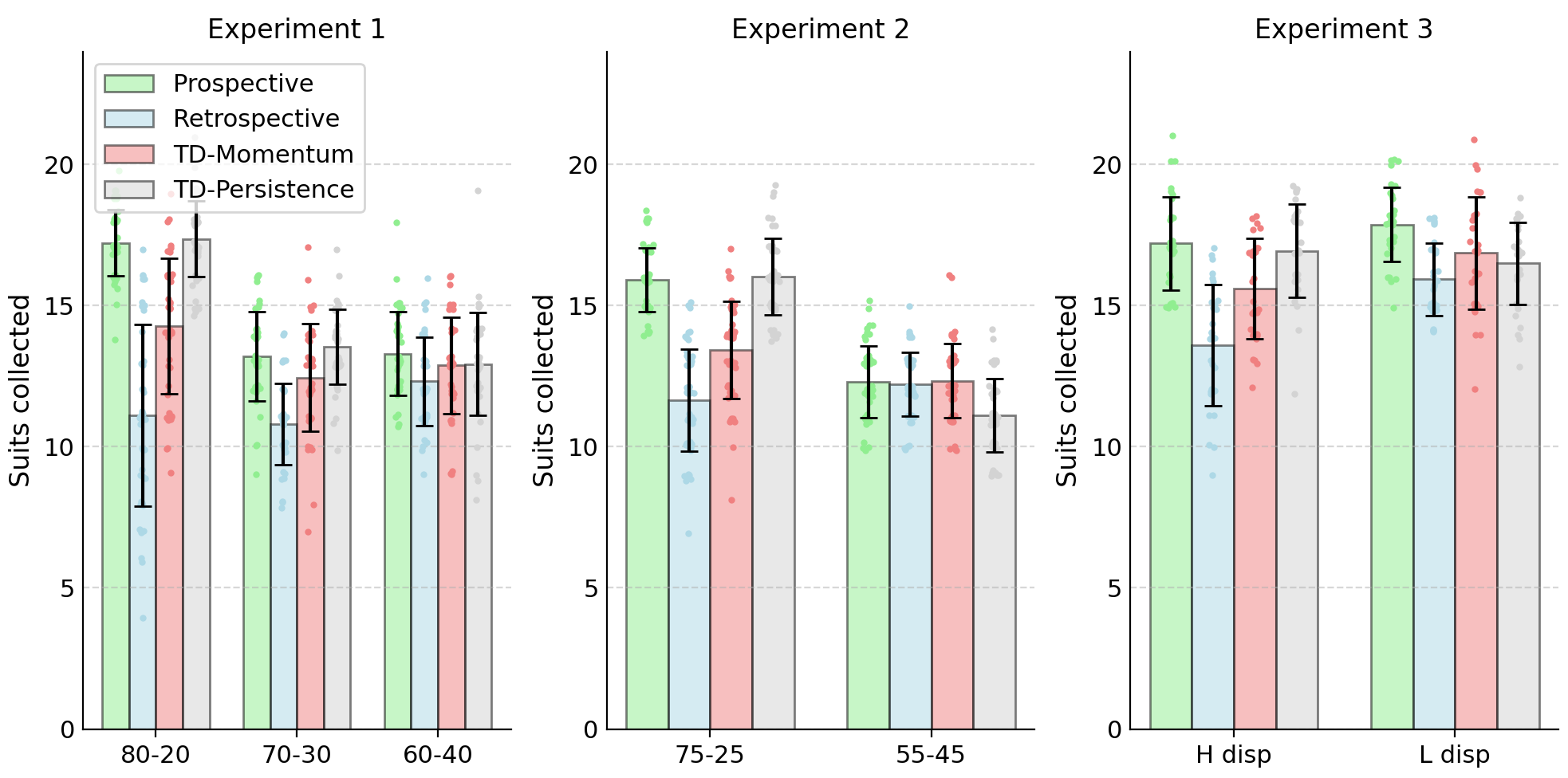

Supplement: S7 Fig — Number of suits collected by each agent broken down by block type when their parameters are optimized for task performance. Prospective and TD-persistence agents perform the best, and the retrospective agent gives the lowest performance. TD momentum performs better than the retrospective agent but is short of the other agents. (TIF) [file pcbi.1013054.s008.tif]

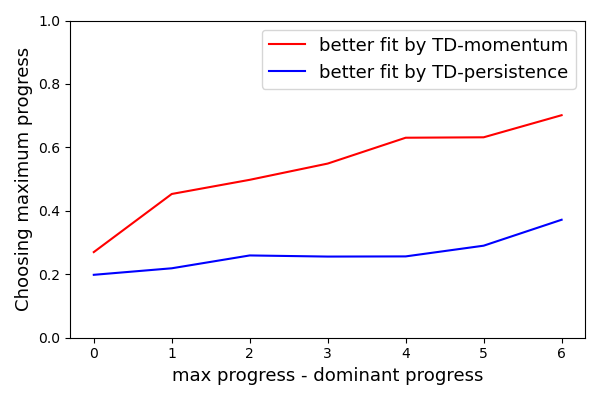

Supplement: S8 Fig — 40% of participants in the version with explicit instructions about the current block type were better accounted for by TD-persistence, with the rest 60% favoring TD-momentum according to BIC criteria. Lower retrospective bias and higher task performance is observed in the group that is better accounted by TD-persistence. Retrospective bias of the participants explained by TD-persistence follows the similar pattern as demonstrated by simulations of the algorithm. (TIF) [file pcbi.1013054.s009.tif]

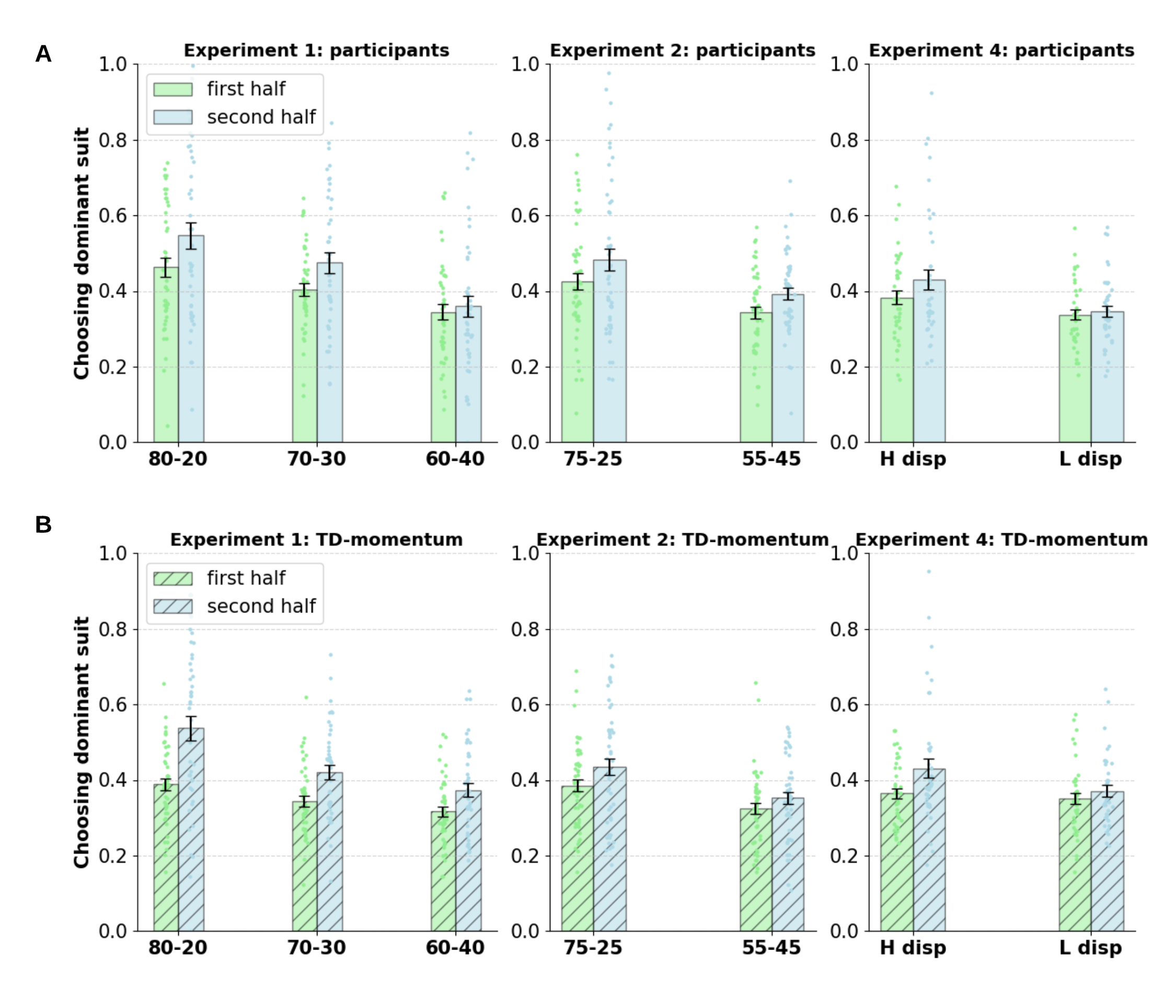

Supplement: S9 Fig — Figure shows the participants’ dominant suit choice probability in the first and second halves of the block across all experiments replicated by TD-momentum. (TIF) [file pcbi.1013054.s010.tif]

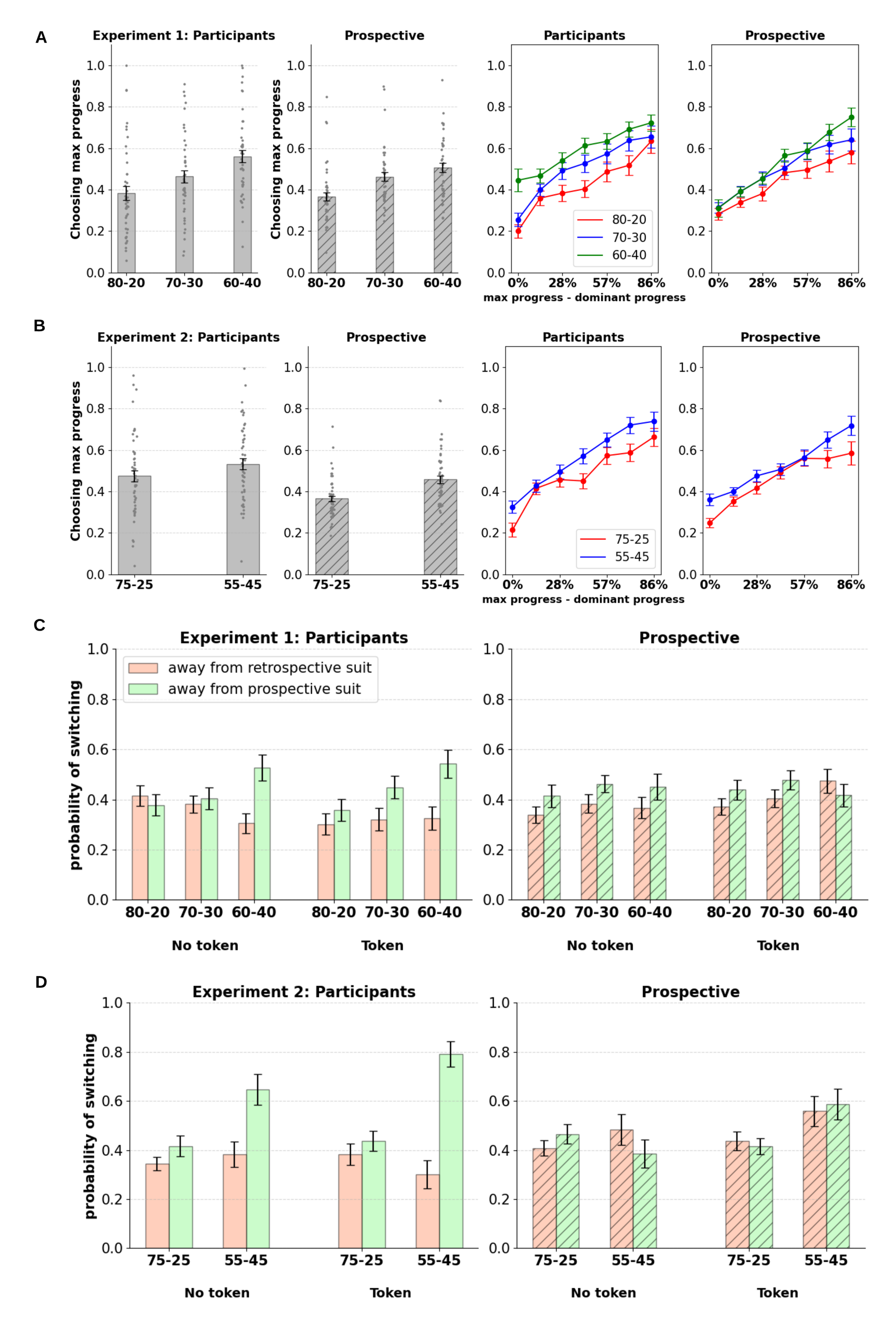

Supplement: S10 Fig — Prospective agent captures general trends in preferences towards maximum progress suit and switching patterns in the task. (TIF) [file pcbi.1013054.s011.tif]

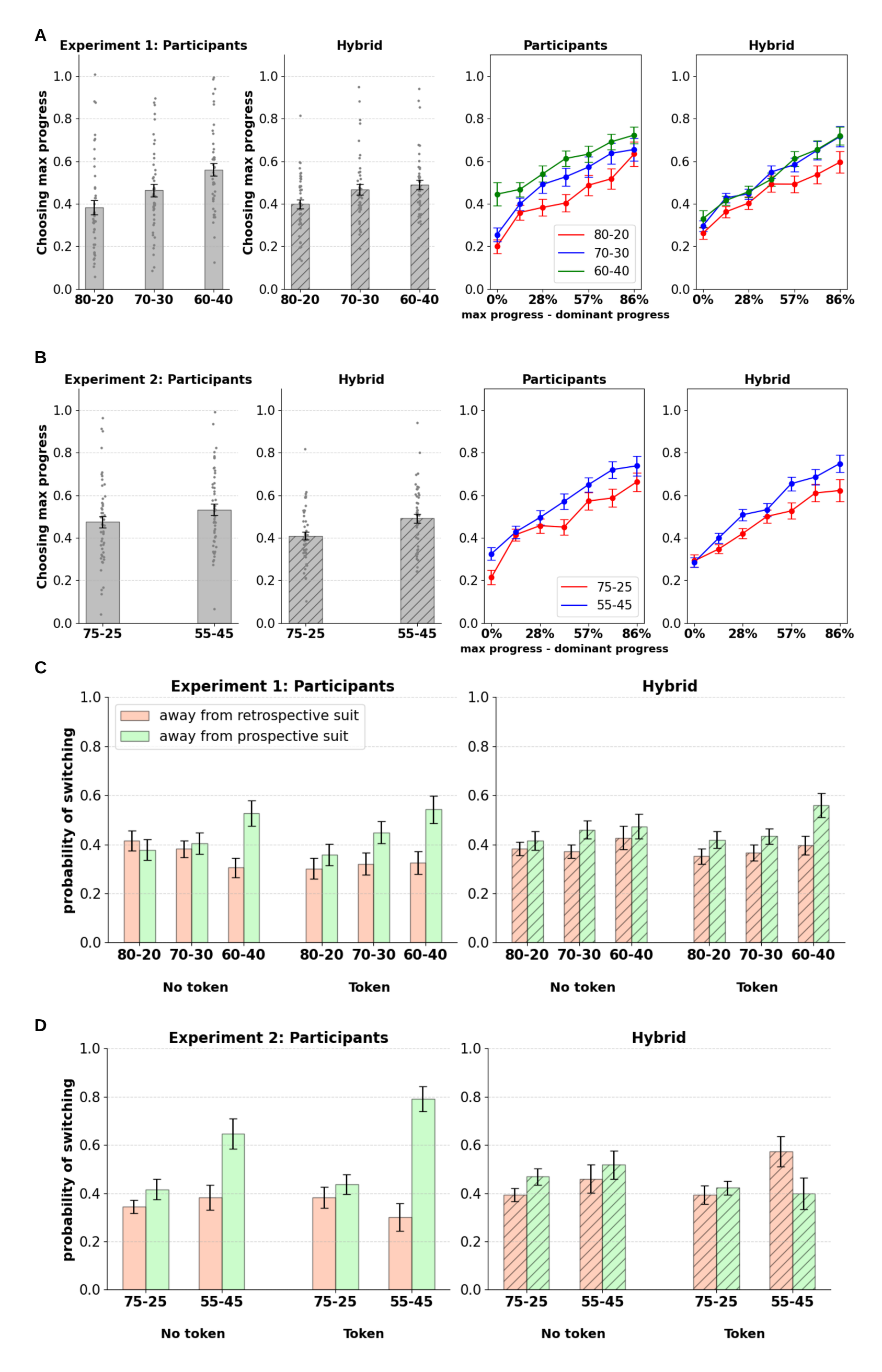

Supplement: S11 Fig — Hybrid agent captures general trends in preferences towards maximum progress suit and switching patterns in the task. (TIF) [file pcbi.1013054.s012.tif]

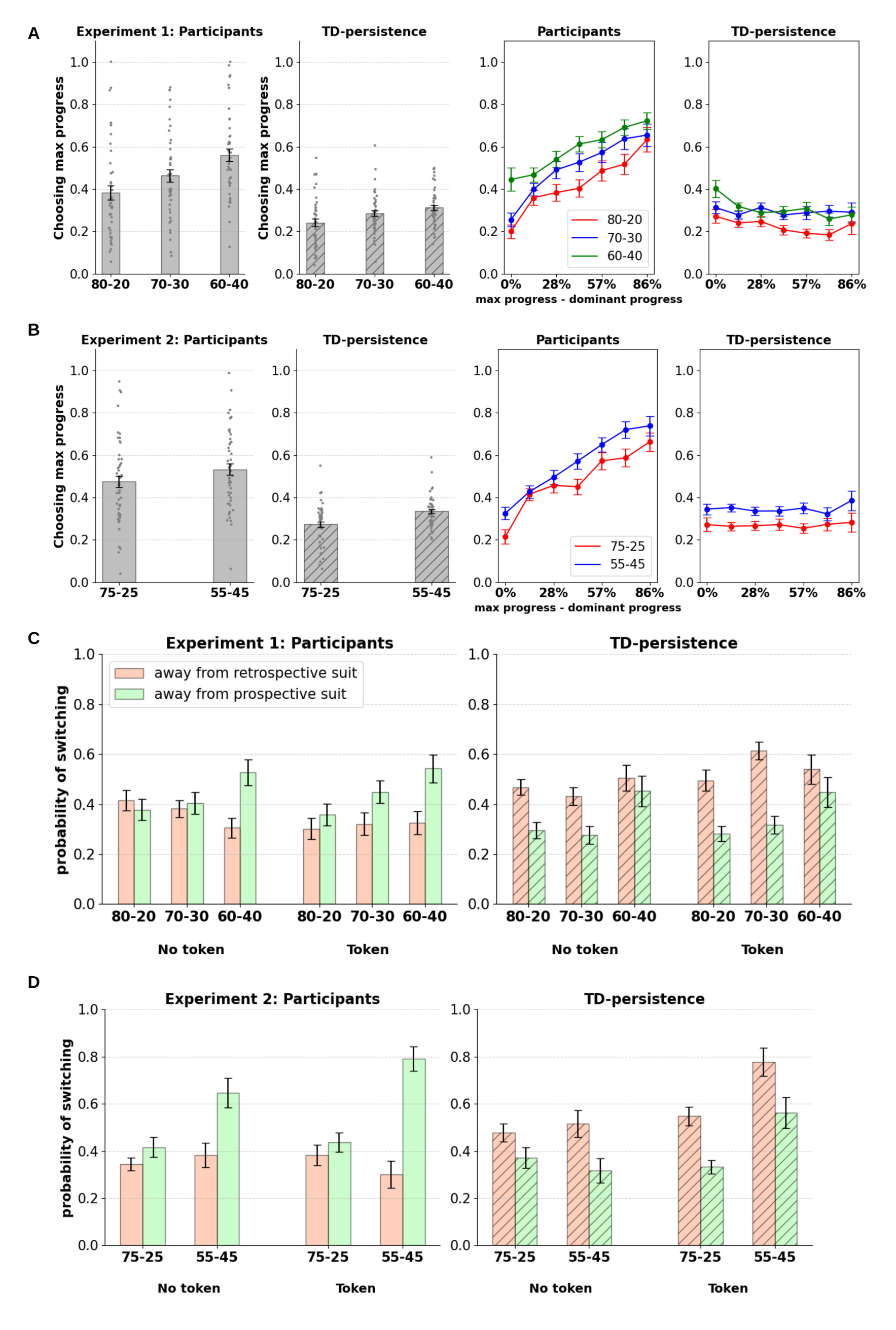

Supplement: S12 Fig — TD-persistence agent does not capture preferences towards maximum progress and also shows increased preference towards switching away from the retrospective agent in contrast to the participants. (TIF) [file pcbi.1013054.s013.tif]

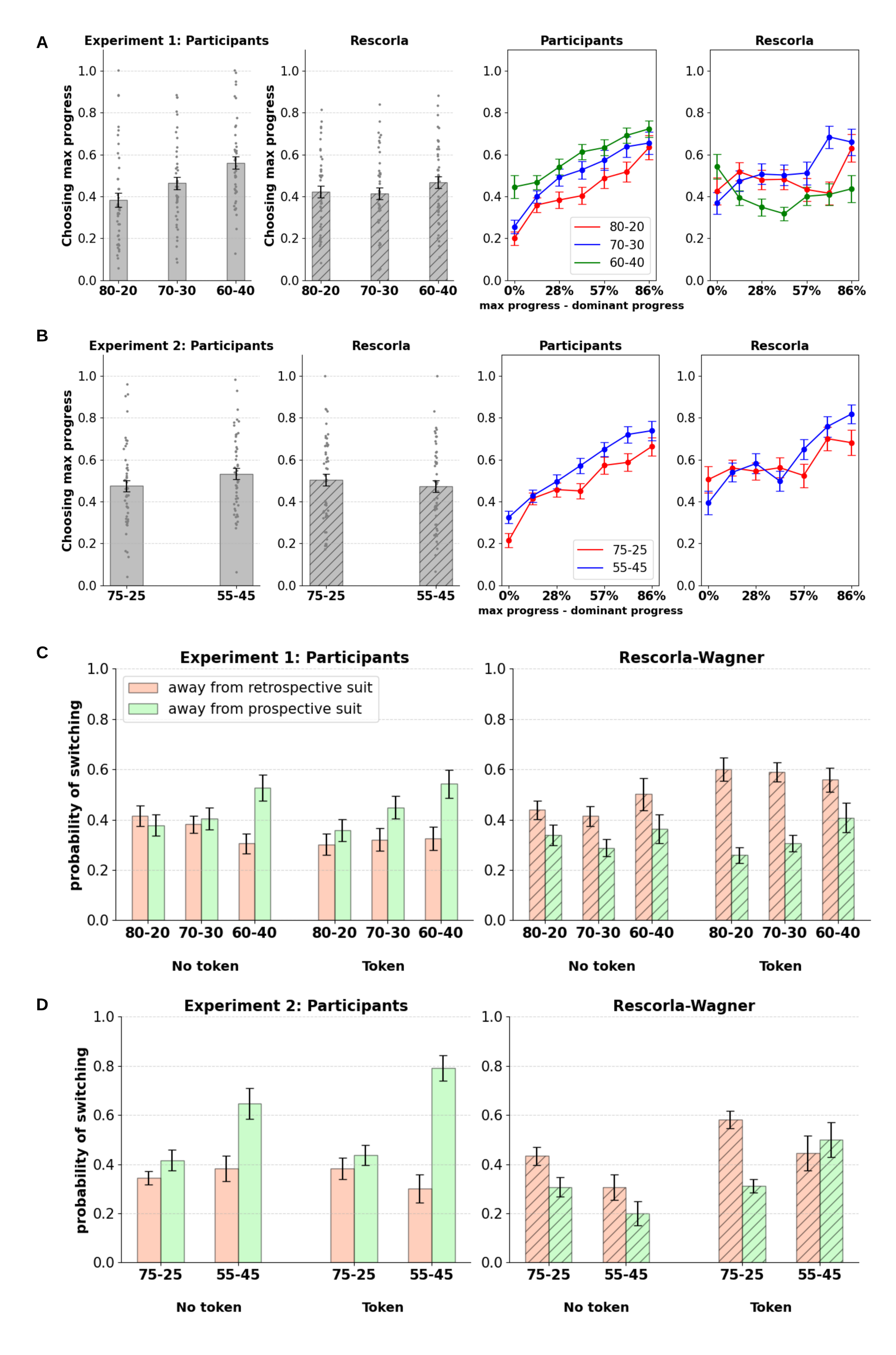

Supplement: S13 Fig — Rescorla-Wagner agent neither captures preferences towards maximum progress nor patterns of switching seen in participants. (TIF) [file pcbi.1013054.s014.tif]

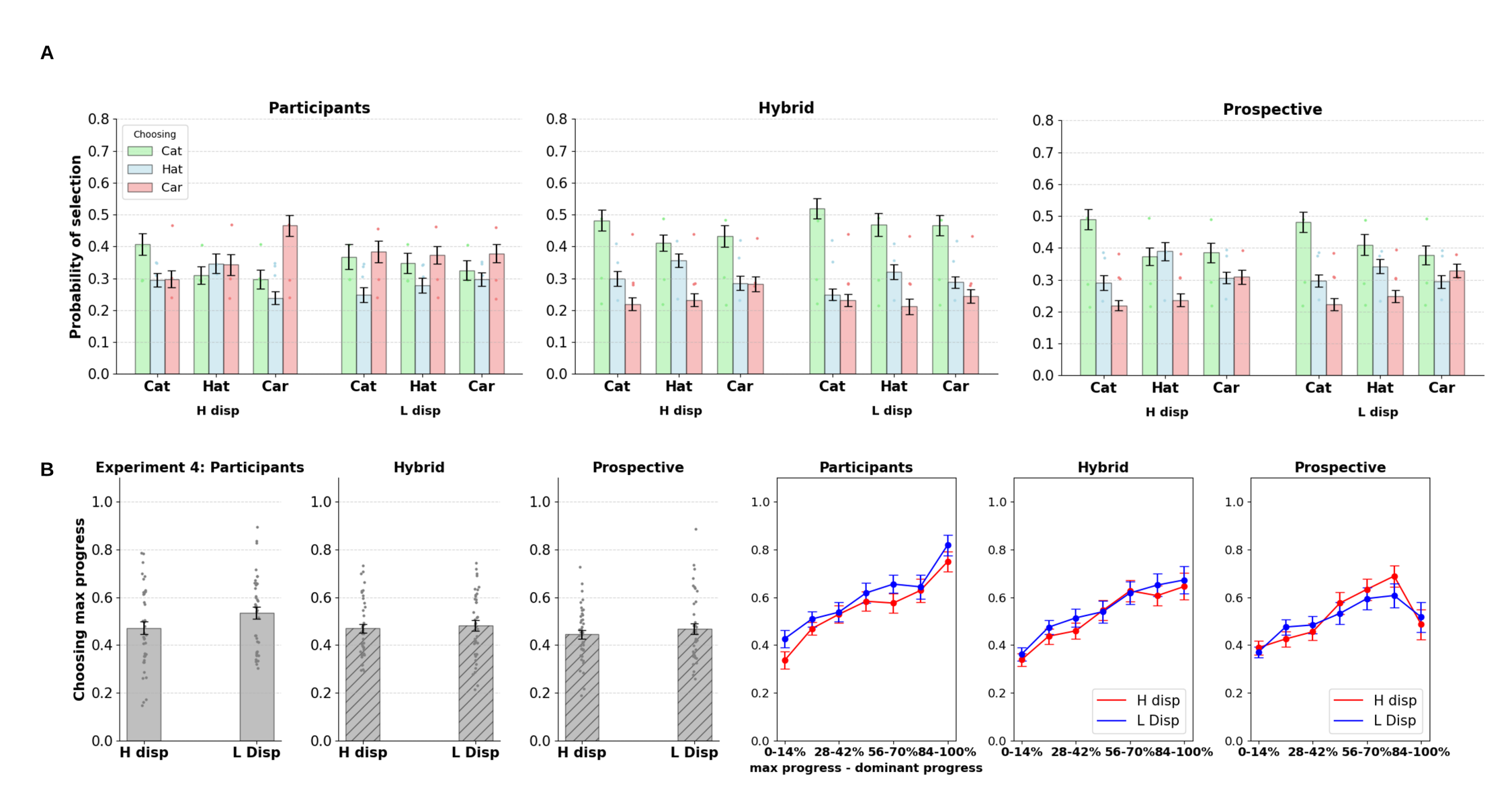

Supplement: S14 Fig — Both hybrid and prospective agents show a preference for the cat suit (smallest target) across conditions. (TIF) [file pcbi.1013054.s015.tif]

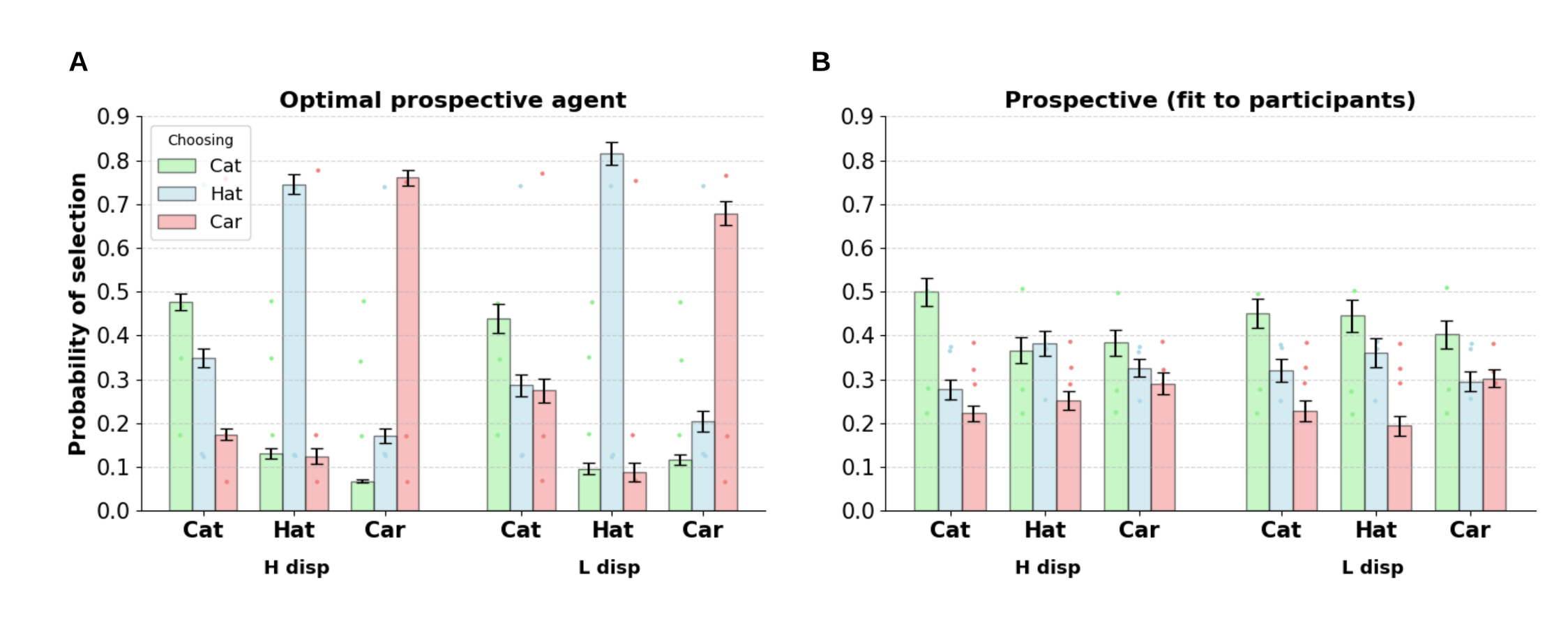

Supplement: S15 Fig — Optimal prospective agent prefers optimal suit in each condition. Prospective agent fitted to participant data displays bias towards the cat suit. (TIF) [file pcbi.1013054.s016.tif]
