# Supplementary material for: Building momentum: A computational account of persistence toward long-term goals
Source: PLoS Comput Biol. 2025 May 14;21(5):e1013054. doi: 10.1371/journal.pcbi.1013054 (PMC12101773; doi:10.1371/journal.pcbi.1013054)
Supplement: Attachment — Submitted filename: PCOMPBIOL-D-24-01340_Review_Pezzotta.pdf [file pcbi.1013054.s017.pdf]

In this manuscript, the authors investigate the tendency of humans to persist in pursuing a goal even when changes in the statistical properties of the environment are such that switching to a different goal would be optimal in terms of long term return.
This tendency is what they term "momentum", in analogy with the concept of inertial motion in physics.
In order to do this, they introduce a novel task in which, at any given time, participants need to commit to one among several (three) possible goals (collecting a target number, or "suit", of tokens of a given type); at any given time, one of the goals can be progressed through at a rate higher than the others (one token type can be collected with highest probability at every round), and its identity changes every few tens of actions.
Completing a goal (suit) requires persistence for a relatively large number of actions, but over this time the identity of the fastest-progressing goal can change. This poses a dilemma: should the participant stick to the goal closest to completion (termed "retrospective"), or should they switch to the new, fastest-completing goal ("prospective")?

The authors show that participants exhibit a bias towards the retrospective choice. The computational device they introduce to quantitative recapitulate results in human behavior is “momentum”, defined as the product between the progress towards a goal and the rate at which progress is made.

The task introduced is novel, the analysis brings out the main points nicely. On the theoretical and modeling side, I have not seen “momentum” defined as the authors do and therefore think it is worth reporting.
However, I think the exposition can be made clearer. For this reason, I do not recommend publication of this manuscript in Plos Computational Biology in the current form, but would consider it if the following points were addressed.

Comments

0. The code, which seems well written, lacks comments. It would be helpful to see more detailed comments at the time of publication.

1. There are too few equations.
This is in part my personal preference, but I do feel that the main problem with the manuscript is the lack of clarity in the mathematical details – especially since it is meant for a theory-inclined audience. In introducing some concepts, the exposition is too verbose and I felt like no equation is either presented in the main text or linked from the Methods when most needed. When the equations are provided, quantities appearing in them are not clearly explained (e.g. Eqs 2-5 at p.13).
I will make more punctual comments below.

2. I feel that the task can be described more clearly. Figure 1 helps a lot, but I find very confusing the description of the relationship between cards and token types (p.3). How do the probabilities come about, from the task design, i.e. are the token probabilities dependent on the

card that one picks, or are the semantic relationships between token type and card only needed for the experimenter to know the goal of the participant? If the former, then it is not clear from Fig.1; if the latter, even more problematic, the performance would not depend on the choice of the cards.
Moreover, task parameters should either be clearly explained in the main text, if important, or left in methods and referenced as details, if not relevant to understanding the paper. For instance, at p.4, we read “Blocks last about 30 rounds”. Does “about” mean that the block duration is random? Presumably, it is important that there are 30 rounds, because that can be compared to how long it takes on average to complete a suit?

3. I would suggest using equations to define what a “retrospective” and a “prospective” suit would be. In determining the prospective suit, one needs to know how the objective function (the discounted return?) is defined. Here the notion of discounting, and the choice made for it, should be discussed more thoroughly (see also comment 6a). We do not see a discount factor until p. 13. Also, at p.5, the authors state that their analysis mainly focuses on rounds where prospective and retrospective choices are different, but that results from remaining rounds are shown for completeness; where is the distinction made, in the main text or in the figures, and where is it not?

4. I have some issues with how “momentum” is introduced and used throughout the text. While reading the manuscript I felt that the actual mathematical definition of momentum in physics was not adding anything to the intuition of what “momentum” means in the manuscript. In fact, for the sake of understanding the authors’ work, Eq. 1 is only qualitative and does not add much to previous verbal description of “momentum”; similarly, it is not clear how momentum is mathematically defined in Eq. 2 – is it perhaps the product of m and v_g? If that is correct, m is not constant, as opposed to the inertial mass in physics (which I believe the name alludes to). The mathematical analogy of “momentum” in goal pursuit with physical momentum therefore breaks down as soon as one puts it in formulas. Another source of confusion for me was that v_g decreases as we get closer to the goal, but m increases in such a way that their product still increases.
For the reasons above, I would refrain from belaboring the analogy with physics beyond one line in the introduction. I do not think one wants to define momentum as “m*v” because that’s how it’s defined in physics, but rather, because that product tells us something about behavior that we want to capture – which has to be better spelled out when it is introduced mathematically, by introducing the equations and letting them speak for themselves.
To that end, I would suggest eliminating the section “Momentum as a key computational variable driving temporally-extended goal persistence”, in favor of a better explanation of the equations in the following “TD-momentum” subsection.

5. The definition of prospective agent and retrospective agent (p. 9-10) is not clear to me from the main text. There are equations in the Methods, but they are not referenced. Also, the first 3 paragraphs of p.10 are vague. What does it mean that the agent operates “under the assumption of a partially observable Markov decision process”? How is the POMDP defined?

What are the states, the observations, the actions etc? How are values estimated? This is crucial, but never shown. Again, we need equations.

6. In relation to temporally-extended goal selection / options and evaluation of delayed rewards in a normative framework for decision making, I feel there are at least two concepts that should be included in the discussion.

a. *Time-inconsistent discounting*. It has been shown that human decision making is better described by hyperbolic discounting, that is not time-consistent (i.e. the ranking of the discounted values of options is not maintained over time), contrary to exponential discounting. One instance in which this may be an alternative/additional mechanism behind participants preferences, is in describing (at least partially) the tendency of the "retrospective" bias as a function of the progress achieved: if the block switch occurs when we are very close to suit completion, the retrospective option may yield little reward (or reward 1 with low probability), but this would still happen quickly compared to the completion of the prospective suit – and therefore we would still choose the retrospective one. Although this is true also with exponential discounting, I would expect this bias to be stronger with hyperbolic one, in closer correspondence with the results from human experiments reported by the authors.

b. *Hierarchical reinforcement learning algorithms*. Attempts have been made to extend the MDP framework in order to account for temporally-extended *options* (see "Temporal abstractions through options" in the RL book by Sutton and Barto), by introducing a hierarchy of actions (see, e.g. Botvinik "Hiearchical reinforcement learning and decision making" (2012)). The important difference between the notion of temporally-extended options, vs temporally-extended goals, is that the former implies a "pre-planning" of a sequence of actions (like flipping the same cards for a fixed number of rounds), while the latter implies re-evaluation of each goal/option at any moment. I think it would be interesting to bring this out.

7. In Fig 3, I feel it would be better to color-code the bars by retrospective/prospective, and group the pairs of bars by token/no-token (so that significant differences would be shown between two adjacent bars, and not spanning between the two groups). Same for Fig 7, 8E, S7 and S8.

8. Fig 6 – and in all model comparison figures. "Score" suggests "the higher the better", but that is not the case for the AIC/BIC scores, that contain a negative log-likelihood – making them into "costs", rather than "scores". Introduce the formulas for the AIC/BIC in the main text. Or clearly indicate to the reader that in Fig.6, "the lower the score, the better the fit".
In Fig.6C it is unclear what the quantity being plotted is.

9. As a general point: all figure legends require more explanation.

10. In Fig.7, I would plot participants and model results in the same graph for easier comparison – e.g. with different shading / dashing

11. In the legend of Fig 4., “Behavior” → “Participants” (the other two are types of agent).

12. Fig 4. Labels in captions are wrong – There is no panel C.

13. Some minor points about wording
    a. “Relegated to the status of sour grapes” (p.2) – is it a common expression? If not, opt for a more immediate and precise one.
    b. “Lucrative” (p,7) → “advantageous”/“preferable” (?)
    c. “Pales in comparison to” (p.10) → “is much lower than”
    d. “Curtailing exploration” → “reducing” (?)
    e. “Interaction with block types” (p.7) — unnecessary technical stats jargon → “dependence on” (?)
    f. “Interacts with the block condition” (p.5) – same as e.
    g. Remove “clearly” from “clearly evident in the” ecc. (p.11)
