# Supplementary material for: Building momentum: A computational account of persistence toward long-term goals
Source: PLoS Comput Biol. 2025 May 14;21(5):e1013054. doi: 10.1371/journal.pcbi.1013054 (PMC12101773; doi:10.1371/journal.pcbi.1013054)
Supplement: Attachment — Submitted filename: PLOS Comp Bio 2024 Building Momentum.pdf [file pcbi.1013054.s018.pdf]

Building momentum: A computational model of persistence in long-term goals – PLOS Comp Bio 2024

In this paper, Aenugu and O'Doherty address the issue of goal persistence under the lens of momentum, a concept borrowed from Newtonian physics that is a function of both mass and velocity. In a novel task, the authors observe that people persist in trying to make progress toward the current goal even when switching to a different goal is optimal. By applying the notion that more force is required to change the movement of an object with high momentum, the authors capture human goal persistence behavior across multiple experiments through a newly introduced computational model. The authors also provide a possible explanation for this suboptimal behavior, which might be well-suited (simple and efficient) for more naturalistic settings.

Goal selection is an important topic that has long been studied but is missing a computational cognitive perspective addressing, testing, and explaining it. I enjoyed reading this article and found the task interesting, the analyses thorough, and the momentum perspective novel and stimulating. However, I have some concerns and clarifications about the modeling approach that the authors should address. In particular, the authors should evaluate and address potential confounding explanations form competing models and artifacts from the experimental design. Moreover, the authors should take further steps to validate the experimental procedure and their interpretation of the results. In addition, I found the contextualization of the authors' current work and the existing literature insufficient, as it failed to reference many existing, highly relevant published articles. Some clarifications and further information about the methods are also warranted. I list below in more detail the points of major concern that should be addressed to strengthen this submission.

Main questions and comments

I find the authors' work original and their contribution valid. However, I was surprised by how little their study was integrated with the existing literature, including recent and seminal studies.

- The "retrospective" pull effect the authors find here has been extensively studied by several experiments in humans and other animals, where it was addressed as the "sunk cost fallacy" (Arkes et al., 1985 Organ Behav Hum Decis Process; Sweis et al., 2018, Science; Cheng et al., 2023, Cognition; just to name a few examples). However, the authors never mention this phrase, nor do they cite any relevant literature.
- A recent, highly relevant paper presumably escaped the authors' attention. Holton et al. (2024, Nat Human Behav), recently published a study in which they addressed goal persistence bias with a task similar to the authors'. They also provided a computational model of participants' behavior, which they claim is capable of capturing key behavioral signatures observed in human participants. It is necessary for the authors to:
    - Explain how their study differs from Holton et al.'s.
    - State their new contributions clearly.
    - Compare their proposed computational model to Holton and colleagues'.

- Moreover, the concept of momentum per se does not imply the presence of a goal in the definition of velocity. The authors thus make an additional step in making an analogy between goal progress and momentum that should be clearly stated and addressed.

Another important issue to address is the fact that it takes participants a few trials to learn the new outcome contingencies. Even in experiment 3, where participants are told what the new probabilities are, they still need to figure out by trial and error which suit has the highest probability of yielding goal progress. Moreover, if participants are not told that consecutive blocks cannot have the same best suit, they might think the current goal suit is the best one even after being informed of a switch. Therefore, some goal persistence should be expected for the few trials immediately following the switch. If that is the case, participants would also fail to explore alternative options – needing to rely on the lack of successful attempts on the retrospective goal rather than experiencing the potential benefits of switching through exploration. One way to address this potential confound would be to introduce forced trials in the experimental design to ensure proper exploration of the alternatives. However, the authors might also propose ways to exclude this alternative explanation through additional analyses of the data they have already collected.

I have some additional concerns regarding the proposed computational model.

- It is unclear to me which γ was used for the prospective (optimal) model (p. 10). With high levels of delay discounting, might we expect the optimal model to perform more similarly to the participants?
- The authors do not justify the presence of a bias term for each goal in their TD-momentum model (p. 13). More importantly, it is unclear why the bias would need to be updated via the learning rule. Is this mechanism necessary for the model to capture participants' behavior? If so, it would be useful to show this necessity in the supplements (e.g., comparing to a simpler model lacking this term). Moreover, the authors should explain how this term affects their interpretation of momentum and the way it is applied to human goal persistence.
- It is unclear to me how the authors modeled goal selection. If they used information from goal probes, was the assumption that goals remained the same before/after the next goal probe? If they inferred it from participants' choices, how could the model (and associated likelihood) distinguish between an unlikely, yet possible, goal-incongruent action vs. a switch in goal?
- Simulated behavior from any of the models the authors present does not match the participants' behavior well in Experiment 4. The authors recognize this limitation but do not address it. Could the authors explain the mismatch, and propose extensions of their model to address it?

Before diving into plots and analyses of interests, some task validation steps should be performed.

- The authors seem to rely a lot on goal probes. Therefore, it is necessary to understand how consistent responses to probes were with participants' responses in the task.
- It also seems important to address whether/how goal probes might have impacted the participants' choices throughout the task. For instance, the probes might have prompted goal re-evaluation. Alternatively, goal probes might have prompted participants to act consistently with their declarations beyond what they thought was optimal for the task (e.g. to avoid cognitive dissonance), yielding goal persistence effects to suboptimal levels. Were goal switches more/less frequent around goal probes?
- Since switches occur at regular intervals, people might learn the structure of the task over time. Was persistence less strong at the end, compared to the beginning of the task?

The authors also state that "It is more lucrative to switch away from the retrospective suit in high disparity blocks than in low disparity blocks" (p. 7). However, this is not always the case: if the horizon is long enough, it should always be optimal to switch. By contrast, if only a few trials remained in the task, it might be more advantageous to stick to the current goal.

- Were participants aware of how many trials were left in the trial/block?
- Did their behavior change towards the end of the task?

The last section on simulations is useful to test whether the observed suboptimal behavior corresponded to optimal choices in a different, and more naturalistic setting. However, the authors should address (at least in writing) other possible explanations. For instance, Cheng et al. (2023, Cognition) find that humans are biased towards keeping their goals consistent as it helps inform others of one's intentions in social contexts. Therefore, people might be biased toward goal persistence even in asocial settings out of an acquired habit.

The Discussion section failed to mention any existing limitations of the current work, and how they might be addressed by future work.

Additional information about the analyses and methods is also needed.

- The authors perform analyses split by high vs. low progress differences between the prospective and retrospective suits. However, I could not find information about the cutoff between high and low differences, making it hard to interpret related results.
- In the Methods section (p. 25), variables need to be explicitly defined. For instance, I_g only gets defined later on, and M_g never.
- I could not find information about the participants' mean age and SD. Given the wide age range presented in Table 1, the authors might also want to address potential age effects in their results.

Lastly, it would be useful to have an upfront overview of the four experiments and the simulations stated before diving into the main Results section.

Minor corrections

More details and clarifications are needed in the task description in the Results section.

- The number of blocks and trials per block appear in the Methods, but would be helpful to have in the results, too.
- The phrase "to ensure the goal selection is not tagged to stimuli" is unclear, so the design choice does not appear well-justified (p. 3).
- My understanding is that switching from one suit to another does not forgo previously accrued goal progress. If this is correct, it would be helpful if the author state this explicitly (instead/in addition to declaring "no penalty").
- The authors should clarify how a block transition is signalled.
- The phrase "Participants can select between [among] three suits in the task" might mislead a first-time reader into thinking an explicit goal selection is made (p. 5).
- Clarify (here, and in the Methods) exactly which blocks are high vs. low disparity, instead of giving examples (p. 5).

Some corrections need to be applied to figures and/or figure captions.

- In Figure 4, subplot B is mislabelled and a reference to subplot C is missing.
- Consider adding an explicit reference to experiment 3 in Figure 5 or its caption.
- The "Prospective" label is somewhat confusing, as it is unclear whether it refers to the optimal model or the Prospective model fit to human participants (p. 11).

I also have a suggestion for an edit to the Discussion section. On p. 22, the authors state that they informed participants of "the token-outcome contingencies of the current block". This can easily be interpreted as telling participants about which suit was the optimal one in a given block, which is incorrect.

Additional minor corrections and typos are listed below.

- Should citations be compressed? E.g., [2-4] instead of [2],[3],[4]
- Sometimes, the suit name is capitalized in the text, but should instead be lowercase and in italics for consistency (e.g., p. 4, "HAT"). Similarly, "Cat" should be lowercased on p. 18.
- Replace"Participants overall, " with "Overall, participants " (p. 5).
- No comma needed after "closer one to the target" (p. 7).
- Insert "The" before "Persistence in goal selection that we describe here" (p. 8).
- In describing the momentum model on p. 14, the authors state "if there is a unit of progress ($m \rightarrow m + 0.14$)" etc. However, the correspondence between a unit of progress and 0.14 is not true for experiment 4, so the authors might want to provide a more general description.
- Replace "whether TD-momentum provide" with "whether TD-momentum provided" (p. 15).
- Insert "The" before "Dominant token" (p. 24).

- Insert “the” before “state of the goal” (p. 26).
- “Rescorla” and “Wagner“ should be capitalized in “rescorla-wagner” models.
