# Supplementary material for: Building momentum: A computational account of persistence toward long-term goals
Source: PLoS Comput Biol. 2025 May 14;21(5):e1013054. doi: 10.1371/journal.pcbi.1013054 (PMC12101773; doi:10.1371/journal.pcbi.1013054)
Supplement: Attachment — Submitted filename: Goal_selection_manuscript_plos_cb_address_to_reviewers.pdf [file pcbi.1013054.s019.pdf]

Address to reviewers:
Building momentum: A computational model of persistence in long-term goals

Sneha Aenugu, John O'Doherty

November 2024

We thank the editors and the reviewers for taking the time out to read our work and give rigorous and insightful comments on our methodology. We believe their feedback made our latest submission a significantly improved version over the original one. Below, we address reviewers' comments point-by-point. Reviewers' comments are indicated in black, our comments addressing the reviewers are in red, and the changes reflected in the manuscript are indicated in blue.

Reviewer 1

In this manuscript, the authors investigate the tendency of humans to persist in pursuing a goal even when changes in the statistical properties of the environment are such that switching to a different goal would be optimal in terms of long term return. This tendency is what they term "momentum", in analogy with the concept of inertial motion in physics.

In order to do this, they introduce a novel task in which, at any given time, participants need to commit to one among several (three) possible goals (collecting a target number, or "suit", of tokens of a given type); at any given time, one of the goals can be progressed through at a rate higher than the others (one token type can be collected with highest probability at every round), and its identity changes every few tens of actions.

Completing a goal (suit) requires persistence for a relatively large number of actions, but over this time the identity of the fastest-progressing goal can change. This poses a dilemma: should the participant stick to the goal closest to completion (termed "retrospective"), or should they switch to the new, fastest-completing goal ("prospective")?

The authors show that participants exhibit a bias towards the retrospective choice. The computational device they introduce to quantitative recapitulate results in human behavior is "momentum", defined as the product between the progress towards a goal and the rate at which progress is made.

The task introduced is novel, the analysis brings out the main points nicely. On the theoretical and modeling side, I have not seen "momentum" defined as the authors do and therefore think it is worth reporting.

However, I think the exposition can be made clearer. For this reason, I do not recommend publication of this manuscript in Plos Computational Biology in the current form, but would consider it if the following points were addressed.

Comments

1. The code, which seems well written, lacks comments. It would be helpful to see more detailed comments at the time of publication.

   Yes. We are in the process of adding detailed comments to the code and this will be completed by the time of publication.

2. There are too few equations. This is in part my personal preference, but I do feel that the main problem with the manuscript is the lack of clarity in the mathematical details – especially since it is meant for a theory-inclined audience. In introducing some concepts, the exposition is too verbose and I felt like no equation is either presented in the main text or linked from the Methods when most needed. When the equations are provided, quantities appearing in them are not clearly explained (e.g. Eqs 2-5 at p.13).

   We appreciate this feedback and have worked to improve mathematical clarity throughout the manuscript. More clarifications to follow.

3. I feel that the task can be described more clearly. Figure 1 helps a lot, but I find very confusing the description of the relationship between cards and token types (p.3). How do the probabilities come about, from the task design, i.e. are the token probabilities dependent on the card that one picks, or are the semantic relationships between token type and card only needed for the experimenter to know the goal of the participant? If the former, then it is not clear from Fig.1; if the latter, even more problematic, the performance would not depend on the choice of the cards.

   We apologize for the confusion about the task description. We added clarifications to the task description section to make this point clearer in the manuscript.

   Participants can collect tokens for suits playing in rounds. They can accumulate tokens in slots and on each round they can see the progress they make toward different suit types. A sample round is shown in Figure 1C. On each round, participants can choose to collect a specific suit by flipping cards. There are two cards for each suit type, and one of the two is randomly shown on every round (see Figure 1B). For each of the suits, we chose card stimuli that hold a semantic relationship with the suit. For instance, we associated the *basket* and *mat* cards with the *cat* suit, and the semantic relationship is "*places where a cat likes to lie*'. We instructed the participants about the semantic relations between the cards and the suit types, following which they received practice retrieving the suit-card associations before the game began.

   Throughout the game, cards serve to stand-in for the suits, and to collect tokens for a specific suit, participants need to flip the cards associated with the suit. Through this

design, we aimed to eliminate stimulus-related value mapping, which would likely emerge if we were to directly display the suit stimuli (say images of *cat*, *hat*, *car*), and elicit the construct of goals in participants. If say, a participant persists in the goal of pursuing the *cat* suit, they would pick the relevant cards (the *basket* card) when it is available, and the *mat* card when it is not). By making the associated cards appear at random, we mitigated stimulus-elicited persistence effects in our design.

Participants receive tokens upon flipping cards with a given probability. If they receive the token for a suit type in a round, it goes into its designated slots and the participant proceeds to play another round. Once a specific target number of tokens (say seven) of one suit type are accumulated, the participant gets reward for finishing the suit, and the slots for that suit are refreshed for a fresh round of collection. Suit collection is continuous, however, we split the game into blocks of 30 rounds each. In each block, we have a specific probability with which tokens for a particular suit can be collected and flipping either of the cards associated with the suit gives you the token with that probability. Once the block shifts, there is a new probability with which the suit can be collected (see Figure 1D). We instructed the participants of this block structure in the task: specifically that a block lasts 30 rounds and that different suits have different probabilities within a block and that changes when the block shifts. We also emphasized during the instructions that the probabilities are associated with the suit types and that picking either card related to the suit gives the token with the same probability. This is to prevent participants from tracking value associated with specific card stimuli (whether a specific card gives the token more often), and prompts them to place values on the goals (suit types) instead.

Furthermore, we designed blocks in the game so that there is a dominant suit in each block (counterbalanced across all suit types), such that flipping its cards gives tokens with the highest probability (in Figure 1D, the *cat* suit is the dominant one with 0.8 probability in block N). The other two suits have identically inferior token probabilities (both *hat* and *car* with 0.2 probability). Pursuing the dominant suit type on each block gives participants the best chance at success in suit completion. We also varied the probability disparity between the dominant and the inferior suits to see how it modulates goal selection. We expected that the task pressure to select the dominant suit would be higher in the high disparity blocks (where probability difference between the dominant and the inferior suits is high) and would be reflected in participants' goal selection patterns. We instructed the participants of this task manipulation, explaining that within each block there is a specific suit which has the highest chance of token collection. However, we did not emphasize the design manipulation that the dominant token in one block becomes an inferior token in the adjacent block.

We implemented dominant token switches so as to enable us to investigate conflicts in goal selection between retrospective and prospective influences, as this is critical to assess how individuals change their choices of goal in the face of such a conflict.

If a participant pursues a dominant suit in a block and is close to finishing it, and they encounter a block shift when the hitherto dominant suit is no longer dominant, then it is relatively more difficult to make further progress towards its target. A goal conflict arises in these situations, concerning whether to persist in the suit that is close to the target, or to switch over to the new dominant suit. Such scenarios occur frequently during the game, and the optimal strategy is to switch over to the dominant suit in the new block.

We include four experiments using this paradigm. Experiments 1-3 have uniform targets for all suits, with each suit comprising of seven tokens of one kind. Experiment 1 has 18 blocks with 30 rounds each, with three different probability structures: 80-20, 70-30, 60-40. In the 80-20 block (say), the dominant suit is available with 80% chance and the two inferior tokens are available with 20% chance. Experiment 2 has 12 blocks (30 rounds each), with two different block types: 75-25 and 55-45. In both experiments 1 and 2, our instructions are limited in that, within each block, one specific suit has the highest probability, and the probabilities change with each block switch. In experiment 3 (which is a repeat of experiment 1), we gave participants more information about their current block context. Before each block began, we specifically informed them that they are in an 80-20 context, without telling them which specific suit is available with 80% chance (more details on task design and instructions are elaborated in the Methods section). In experiment 4, we employed non-uniform targets for different suits and explored its impact on validating our computational hypothesis.

As for your question, we clarify that the latter part of your surmise is correct (cards are only needed for the experimenter to know the goal of the participant, or rather to induce the participant to think in 'goals'). However, we don't think it is problematic considering the question we are investigating. We extensively deliberated on this question while designing our task. Our primary manipulation is to create block shifts where a viable goal in one becomes not viable in the next. We wanted to maximize block shifts while also maintaining a reasonable block length (30 rounds) to let participants build some persistence within a block. So estimating an optimal goal within a block should be relatively easy, with a few rounds of exploration (pick either card related to the goal suit in a round). If we introduced cards with different probabilities, estimating which goal is valuable within a block would be tricky, as cards need to be tracked as well. This would add considerable noise to the goal-estimation process, as exploration would be tied to the cards as well in this alternative design manipulation.

4. Moreover, task parameters should either be clearly explained in the main text, if important, or left in methods and referenced as details, if not relevant to understanding the paper. For instance, at p.4, we read "Blocks last about 30 rounds". Does "about" mean that the block duration is random? Presumably, it is important that there are 30 rounds, because that can be compared to how long it takes on average to complete a

suit?

We apologize for the lack of clarity here. In fact each block lasts exactly 30 rounds. We highlighted that in the revised manuscript.

5. I would suggest using equations to define what a "retrospective" and a "prospective" suit would be. In determining the prospective suit, one needs to know how the objective function (the discounted return?) is defined. Here the notion of discounting, and the choice made for it, should be discussed more thoroughly (see also comment 6a). We do not see a discount factor until p. 13.

   We appreciate this suggestion. We agree that the notion of discounting should be introduced earlier in the manuscript. We have now done so, which we believe strengthens the narrative of the manuscript. We combined the two sections ("Participants primarily choose between prospective and retrospective suits", and "Participants demonstrate a retrospective bias in temporally-extended goal selection") into a revised section "Participants demonstrate a retrospective bias in temporally-extended goal selection" which is reproduced below. We introduced the prospective agent which prioritizes faster suit completions from a myopic standpoint by focusing on the immediate reward delivered at the next closest suit completion rather than optimizing for the total discounted returns accumulated at the end of the task.

   We added a new figure 2C (goal selection patterns), and revised figure 3 (switching patterns) to incorporate algorithmic definitions of 'prospective' and 'retrospective' suits.

Participants demonstrate a retrospective bias in temporally-extended goal selection

We analyzed goal selection patterns in human participants as influenced by the current context (suit probabilities in a block) and slot configuration. In a given round, participants can choose to collect the dominant suit (with the highest probability of receiving tokens), or chose to pursue the suit with the most progress (max progress suit) as per their slot configuration. In Figure 2A, we show the participants' probability of selecting cards related to the dominant suit, max progress suit, and the third suit. Participants preferred to select either the dominant or the max progress suit across all conditions, picking the remaining suit well below chance level. This indicates that the primary conflict in goal selection is between the accrued goal progress and the current rate of goal progress.

Moreover, the preference towards the dominant/ max progress suit shows an interaction with the block condition. Participants show increased preference towards the dominant suit, when the disparity between different suits is large (80-20 / 75-25 conditions) and reduced preference when the disparity is small (60-40/ 55-45 conditions), indicating sensitivity to the current rate of progress (repeated measures

ANOVA; experiment 1: F(2, 86)=20.23, $p < 0.00001$, experiment 2: F(1, 49)=17.32, $p < 0.00001$). Likewise, preference towards the max progress suit increases with decreases in disparity between the suits (repeated measures ANOVA; experiment 1: F(2, 86)=19.67, $p < 0.00001$, experiment 2: F(1, 49)=7.1, $p < 0.01$). However, across all conditions, participants preferred to pick the max progress suit over the dominant suit, indicating an increased preference for the accrued progress over the current rate of progress.

Figure 2A provides a partial examination of choice patterns, highlighting rounds where the dominant suit and the max progress suit are separate. However, in a fraction of rounds, the dominant suit is the same as the max progress suit, because maximum progress is made towards the dominant suit. In 62% of the rounds in experiment 1, and 63% of the rounds in experiment 2, the dominant suit is separate from the max progress suit while in the rest of the rounds the two options converge. We noted that in the rounds where the dominant and the max progress suit are the same, both the accrued progress and the current rate of progress for the suit are high, mitigating the conflict in goal selection. In those rounds, participants' predominantly chose to the pursue the dominant (or the max progress) option, picking the other two options occasionally and well below chance level (see supplementary Fig S1).

Limiting our analyses to the rounds where the dominant and max progress suits are separate (with a goal selection conflict), we further analyzed how progress difference between the two suits influence goal selection. Figure 2B shows the probability of choosing the max progress suit as a function of the difference in progress between the two suits. We show that participants' preference towards the max progress suit monotonically increases with the increasing progress difference between the suits. This preference is highest when the max progress suit is at 86% progress (6/7 tokens completed), and when the dominant suit has (0/7) tokens collected. The three curves split by block condition in Figure 2B also indicates sensitivity to relative suit probabilities, with the 60-40 monotonic curve lying above the 80-20 curve.

The pattern of increased preference towards accrued progress in human participants is not necessarily suboptimal. If a suit is close to completion, it might be optimal to persist with it and get a reward sooner than switch to the dominant suit that might have a high rate of progress but an equally high trials-to-completion estimate. In fact, humans are known to prefer sooner rewards over later ones, a tendency owing to incorporating a discount factor in estimation of reward value incurred over a delay [4]. We investigated whether participants' goal selection patterns are optimal under this assumption.

We constructed a prospective agent that prioritizes faster suit completion and reward accumulation. The agent, rather than optimizing for discounted returns accumulated at the end of the task, focuses on the immediate reward delivered at the end of suit completion. The agent, thus, places the highest values on goals that have the lowest estimates of trial-to-completion. To estimate values, the agent needs to maintain

beliefs about the current probability estimates of various suits and roll out expectations into the future until the termination condition (collected 7 tokens) is reached. We constructed the agent as a partially observable Markov decision process, with the current slot configuration as the observed state, selection of cards as actions, and an estimate of the current suit probabilities as the belief state. The agent updates the belief about token-outcome probability of a suit using a delta learning rule,

$$M_g^{(t)} = M_g^{(t-1)} + \alpha(I_g^{(t)} - M_g^{(t-1)}) \tag{1}$$

where $M_g(t)$ is the belief at time t, and $I_g(t)$ is the indicator function of the token outcome (1 if token is received in a round), and α is the learning rate. Using the estimated belief probability, the prospective agent rolls out expectations one step into the future, for two scenarios: receiving a token with the probability M_g, and moving one token closer towards the target ($s_g^{(t)} + 1$), and not receiving the token with the probability $1 - M_g$, and staying in the same slot state ($s_g^{(t)}$). Rolled out future states are discounted by a factor of γ.

$$Q_g\big(s_g^{(t)}\big) = M_g \gamma Q_g\big(s_g^{(t)} + 1\big) + (1 - M_g)\gamma Q_g\big(s_g^{(t)}\big) \tag{2}$$

where $Q_g(t)$ is the value of the goal suit g, $s_g(t)$ is the current slot state of suit g. Specifically $s_g(t)$ is a counter for goal progress, with the above recursive estimation terminating when the current state reaches the target (say 7 tokens). Recursion is also terminated with a horizon of 20 steps.

The prospective agent, as defined above, can favor pursuing the maximum progress suit over the dominant suit. When the maximum progress suit is close to completion (say 5 out of 7 tokens), the agent has to unroll fewer steps ahead to receive rewards compared to the dominant suit that is further away from the target (say 2 out of 7 tokens). The prospective agent, however, is sensitive to block conditions — when placed in the 80-20 block, the chance of collecting two additional tokens with a 20% chance is lower than that of collecting 5 tokens with an 80% chance, and the agent prefers to pursue the dominant suit.

We optimized the parameters of the prospective agent (parameters and action selection process documented in the Computational modeling section of Methods) for task performance (total number of suits completed). The optimal discount factor for this agent is γ is 0.95 for experiment 1 and 0.93 for experiment 2. The prospective agent, thus optimized for the task, outperforms human participants significantly (see Figure 4; performance distinctions elaborated in a later section).

For each slot configuration seen by human participants, we computed the response exhibited by the prospective agent and investigated the extent of the participants' deviation from this response. For generating the prospective responses, we assumed

the true state of suit probabilities as the agent's belief (not accounting for learning effects). We define the suit selected by the prospective agent as the *prospective suit* and the suit with the maximum progress in a round as the *retrospective suit*. Figure 2C provides a revised analysis of Figure 2A, indicating the goal selection patterns for choosing between the prospective suit, the retrospective suit, and the third suit (when the prospective and retrospective suit identities are different). The revised goal selection patterns show a similar trend to that shown in figure 2A, with the preference towards the retrospective suit being higher than that of the prospective suit across all conditions. Moreover, the prospective suit shows the same interaction profile with the block condition (repeated measures ANOVA: Experiment 1: F(86)=7.04, $p < 0.001$; Experiment 2: F(49)=17.79, $p < 0.0001$), and likewise with retrospective suit (Experiment 1: F(86)=2.14, $p = 0.12$; Experiment 2: F(49)=8.88, $p < 0.005$). The proportion of rounds where there is a goal selection conflict in the current classification (prospective suit -retrospective suit) is now dropped to 40% on average across all conditions (as opposed to 60% previously in max progress suit - dominant suit classification), indicating that a fraction of max progress choices are indeed prospective in nature after accounting for discounting effects. Supplementary Fig S1 shows preferences when the prospective and the retrospective suit identities are the same (when the prospective agent prefers the maximum progress option) with participants predominantly choosing the prospective/ retrospective option devoid of the goal selection conflict.

In summary, the patterns of goal selection in human participants reveal a retrospective bias for accrued progress in the task. Further quantifying the extent of this bias, Figure 2D shows the overall proportion of dominant suit choice in participants contrasted with that of the task-optimized prospective agent. We also split the choices as occurring in the first and the second halves of blocks (averaged over all blocks) to account for any learning effects. Both participants and the prospective agent show learning effects within blocks with the proportion of dominant choice increasing in the latter half of the block. But participants' dominant choice remains significantly lower than that of the prospective agent in both halves of blocks, accounting for suboptimal human performance in the task (elaborated in Figure 4). Lowered learning rates and increased discounting in participants could potentially account for suboptimality in task performance in humans, which we consider to be a potential account for the goal selection patterns seen in humans. This account is tested in later sections by fitting the prospective agent to participants' choices. Furthermore, there are no significant effects of learning or performance shifts across blocks. Supplementary Fig S3 traces the proportion of dominant choice across blocks where we find no significant changes in the selection effects as the task progresses.

Also, at p.5, the authors state that their analysis mainly focuses on rounds where prospective and retrospective choices are different, but that results from remaining rounds are shown for completeness; where is the distinction made, in the main text or in the figures, and where is it not?

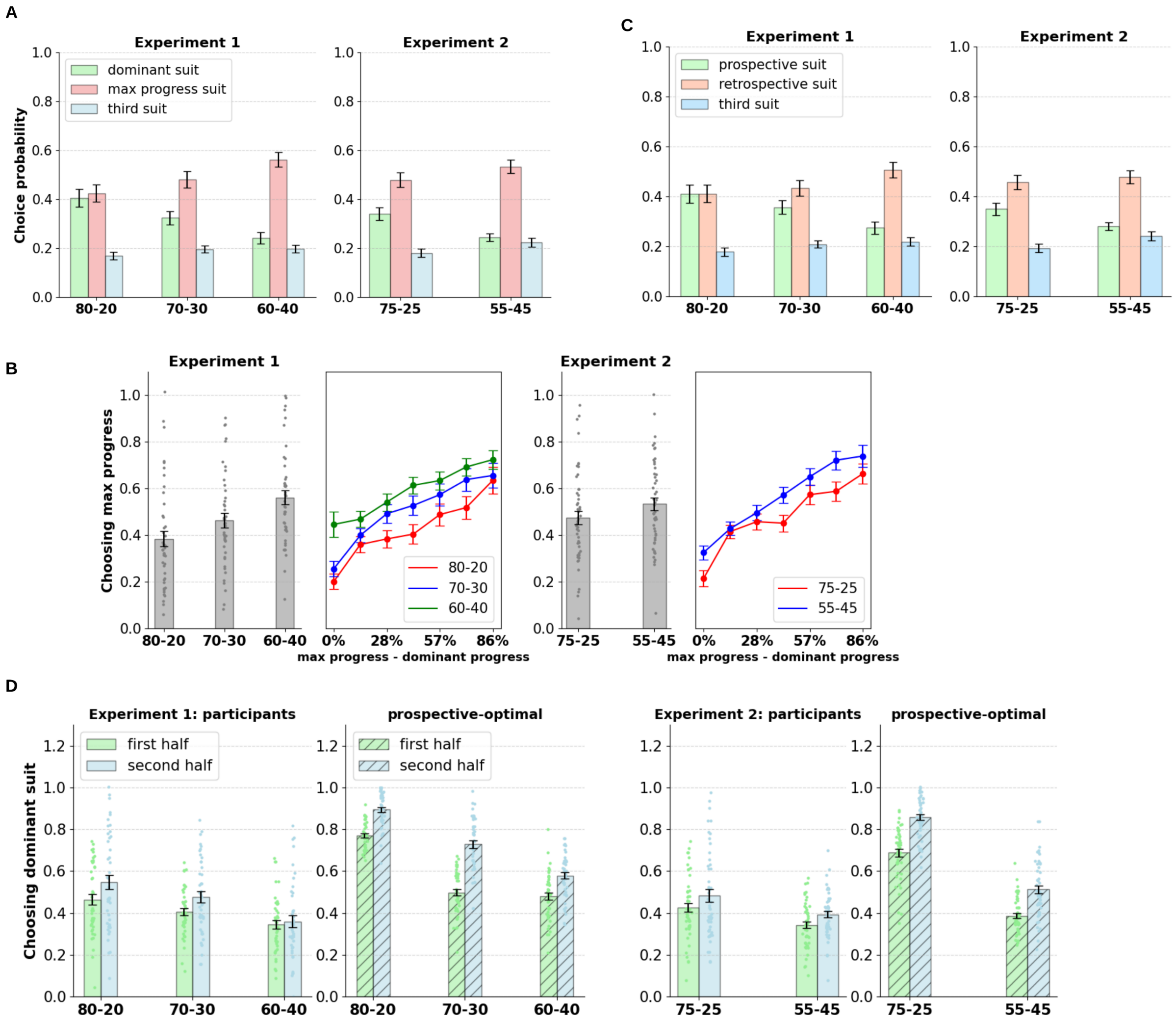

Figure : 2: **A. Participants show a preference for the progress accrued over the current rate of progress.** Participants primarily chose between the dominant suit (the one with the highest probability within the block) and the max (the one with the greatest progress towards the target), while choosing the third option relatively infrequently. **B.** The proportion of times participants chose the max progress suit over the dominant suit (when the two diverge) is broken down by block type. The proportion of retrospectively-biased choices increases with the difference in progress between the dominant and max progress suits. **C. Participants show a preference for the retrospective suit over the prospective suit.** Participants chose the retrospective suit (with greatest progress) over the prospective suit (optimal choice prescribed the task-optimized prospective agent. **D. Learning effects within blocks.** Figure shows the dominant suit choice probability in the first and second halves of the block (15 rounds each). The proportion of dominant choices increases in the second half indicating learning effects within blocks. The choice patterns are contrasted with that of the task-optimized prospective agent indicating the presence of a suboptimal retrospective bias in goal selection.

The figures included in the main manuscript correspond to the rounds where there is a goal selection conflict. When the prospective and the retrospective suits are the same, both accrued progress and the current rate of progress are high, mitigating conflict and favoring goal selection toward the prospective suit. We presented choice patterns in those rounds in the supplementary. We made that point clear in the revised section.

6. I have some issues with how "momentum" is introduced and used throughout the text. While reading the manuscript I felt that the actual mathematical definition of momentum in physics was not adding anything to the intuition of what "momentum" means in the manuscript. In fact, for the sake of understanding the authors' work, Eq. 1 is only qualitative and does not add much to previous verbal description of "momentum"; similarly, it is not clear how momentum is mathematically defined in Eq. 2 – is it perhaps the product of m and v_g? If that is correct, m is not constant, as opposed to the inertial mass in physics (which I believe the name alludes to). The mathematical analogy of "momentum" in goal pursuit with physical momentum therefore breaks down as soon as one puts it in formulas.

   We were drawn to the Newtonian interpretation of momentum, as it provides an account of the physical manifestation of persistence and inertia. An object that is either heavy (high m) or moving at a high speed (high v) has high momentum and offers high resistance to efforts to change its state, measured by the force needed to bring it to a state of rest. Likewise, we claim that the two factors, accrued progress (m) and current rate of progress (v), influence persistence in cognitive states. While it is intuitive to map the current rate of progress to velocity, we agree that the analogy of accrued progress with mass might be less intuitive. However, if we take a speculative leap, accrued progress/ commitment towards a goal in the brain could result in a build up of neurotransmitters or other intracellular messengers in its service, which could provide a direct analogy with the physical construct of mass. As for the objection that mass is constant in our physical world, we point out that mass is only constant in a closed system. For instance, a rocket launched into space ejects fuel and its mass decreases throughout its flight. While the brain as a whole could be a closed system, parts of the brain could still reorganize its configurations (synaptic plasticity, neurotransmitters, and the like) in service of goals.

   However, a deeper dissection of the analogy above is too speculative to be included in the manuscript. Hence, we proceed to add the following disclaimer that our interpretation of physical momentum extends only as an abstraction for formulating persistence in cognitive states.

   The analogy of goal momentum with the physical interpretation of momentum goes only so far as it comprises of two factors, accrued progress (analogous to behavioral mass), and the current the rate of progress (analogous to velocity) that creates resistance to change in valuation. A longer history of reinforcement with an option (accumulated behavioral mass) and a higher current rate of reinforcement both increase

momentum and facilitate persistence with an option. This analogy was previously employed in the definition of behavioral momentum [7], where the rate of free operant responding in the presence of a stimulus is likened to the velocity of a moving body, and the resistance to changing the response is likened to its inertial mass. We, further, extend this metaphor to include extended goals, where the momentum of the goal in reaching its desired set point determines its value.

7. Another source of confusion for me was that v_g decreases as we get closer to the goal, but m increases in such a way that their product still increases.

   We apologize for the confusion. v_g is an estimate of current rate of reinforcement and need not necessarily decrease closer to the goal. It could decrease when the step size of unit progress is low and/ or the discount factor is low. But it could also increase steadily as one gets closer to the goal when the step size and the discount factor is high. Likewise, momentum need not always increase with progress. To demonstrate this concept, we introduced simulations of TD-momentum value estimates as modulated by the discount factor. See the reworked TD-momentum section below.

TD-momentum

We use the aforementioned conceptualization of momentum — a product of progress accrued and the speed of progress — as a measure of goal value.

$$Q_g\left(s_g^{(t)} = m^{(t)}\right) = v_g^{(t)} m^t + b_g^{(t)} \tag{3}$$

where Q_g is the estimate of the goal value of a suit g in a specific slot configuration state $s_g^{(t)}$, m is fractional progress made towards the goal at a given instant t, v_g is the parameter for the current rate of progress, and b_g is a goal-specific bias g.

We formulated an algorithm — TD-momentum — to facilitate updating of estimates of momentum for different goals. Here, the product of the progress accrued and the rate of progress serve as a function approximator for goal value with speed as a free parameter and progress as an indicator of the state of the goal [11]. The estimation of speed is done through the temporal difference learning rule — with its value updated through a gradient descent on the prediction error of the expectation of receiving a token in the next round (and progressing one step ahead).

The temporal difference error (δ) is estimated as follows:

$$\delta = \gamma Q_g\left(s_g^{(t+1)}\right) - Q_g\left(s_g^{(t)}\right) \tag{4}$$

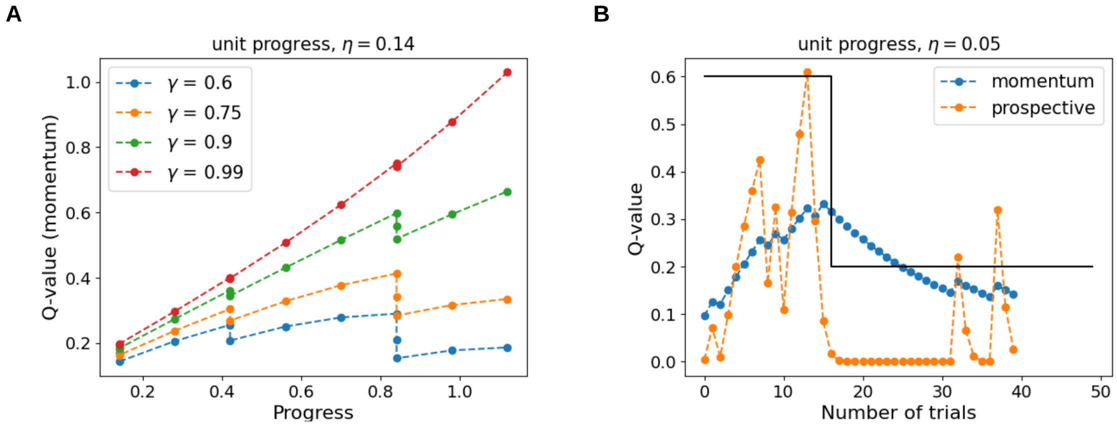

Figure : **A, B. Features of the TD-momentum algorithm. A.** Relation of TD-momentum value estimates with the progress is determined by the discount factor, step size of progress, and also the probability of making unit progress. In the simulations, probability of unit progress is 0.6 and step size of unit progress is 0.14. **B.** Comparison between the value computations from prospective and TD-momentum algorithms. The black line indicates the shift in probability of unit progress from 0.6 to 0.2 after 15 trials. TD-momentum provides stable value estimates as opposed to the prospective model which exhibits fluctuations in response to individual reinforcements. Step size of unit progress is 0.05, learning rate is 0.4, and γ is 0.9 for both algorithms.

TD-momentum parameters v_g and b_g are updated with a learning rate α through a gradient descent on the value function Q_g.

$$v_g^{(t+1)} = v_g^{(t)} + \alpha\delta \frac{dQ_g^{(t)}}{dv_g^{(t)}} \tag{5}$$

$$= v_g^{(t)} + \alpha\delta m^{(t)} \tag{6}$$

$$b_g^{(t+1)} = b_g^{(t)} + \alpha\delta \tag{7}$$

The goal-specific bias term (b_g) is included to ensure that the goal value is updated even when the progress is at $m = 0$. When one chooses the goal when its progress is zero and repeatedly fails to achieve outcome, its value is depreciated through the bias term. The bias term also ensures goal value is retained when the goal target is accomplished (upon which the progress m resets to zero), and can increase the chance of choosing the same goal again even though the value component from the term $v_g m$ is zero.

Here are the key features of momentum computations for goal valuation.

(a) **The relation between goal momentum and fractional progress is modulated by the discount factor, the step size of progress, and the current rate of progress**

If no unit progress is made in a round, $s_g^{t+1} = s_g^t = m$, δ is always negative for $\gamma < 1$.
If a unit progress (η) is made in the next round, $s_g^{t+1} = m + \eta$.
Assuming no goal-specific bias,

$$\delta = \gamma Q_g\big(s_g^{(t+1)}\big) - Q_g\big(s_g^{(t)}\big) \tag{8}$$

$$= \gamma(m+\eta)v_g - mv_g \tag{9}$$

$$> 0 \iff \gamma > \frac{m}{m+\eta} \tag{10}$$

With unit progress (η), prediction errors (δ) are positive if and only if the condition $\gamma > \frac{m}{m+\eta}$ is satisfied (equation 12).

For instance, given $\gamma = 0.8$, a unit progress from $m^{(t)} = 0.2$ to $m^{(t+1)} = 0.25$ will yield a positive prediction error, while a unit progress from $m^{(t)} = 0.9$ to $m^{(t+1)} = 0.95$ yields a negative prediction error. Therefore, for a fixed γ, a small enough unit of progress can result in negative updates for speed when fractional progress (m) is close to 1. This lends an account for the phenomenon of diminishing marginal utility [8], whose salient feature is that the marginal utility of a unit increase in good decreases as its quantity increases.

When no progress is made in a round $s_g^{(t+1)} = s_g^t$, and $\delta = \gamma Q_g\big(s_g^{(t+1)}\big) - Q_g\big(s_g^{(t)}\big) < 0$, updates for the speed of progress are always negative. On the other hand, if there is a unit progress ($m \to m+\eta$) made in a round, the updates to the speed term are positive, but only if $\gamma > \frac{m}{m+\eta}$. Thus TD-momentum's value estimates need not be monotonically increasing with progress, its relation to progress is determined by the discount factor γ, the step size of unit progress η, and the current estimate of rate of progress (v_g). Figure 6A shows demonstration of this feature of momentum computations. We simulated TD-momentum value estimates for several discount factors, with a step size of unit progress of 0.14, and probability of making unit progress as 0.6 (binary 0s and 1s drawn with probabilities 0.4, 0.6 respectively). We note that for $\gamma < 0.9$, TD-momentum exhibits diminished value with increasing progress when the fractional progress is close to one.

However, for most participants in our task, TD-momentum γ estimates through behavioral fits indicate a discount factor > 0.9, so non-monotonic behavior with increasing progress is not observed in the task. However, we expect that with sufficiently lowered step sizes of progress, TD-momentum can predict diminished utility closer to goal completion.

(b) **Goal momentum provides stable value estimates against fluctuations in reinforcements**

We contrast TD-momentum's value estimates with that of the prospective agent in Figure 6B. In the simulation, we introduced a shift in probability of unit

progress from 0.6 to 0.2 after 15 rounds of the task. We note that the value estimates from the prospective agent exhibits fluctuations corresponding to individual reinforcements of unit progress, whereas momentum builds up value steadily and gradually decays when encountered with a shift in probability. We believe, this retention of stable value estimates enables persistence beyond a context-shift which could facilitate sustained commitment toward long-term goals.

For the reasons above, I would refrain from belaboring the analogy with physics beyond one line in the introduction. I do not think one wants to define momentum as "m*v" because that's how it's defined in physics, but rather, because that product tells us something about behavior that we want to capture – which has to be better spelled out when it is introduced mathematically, by introducing the equations and letting them speak for themselves. To that end, I would suggest eliminating the section "Momentum as a key computational variable driving temporally-extended goal persistence", in favor of a better explanation of the equations in the following "TD-momentum" subsection.

We merged the section "Momentum as a key computational variable driving temporally-extended goal persistence" into the section "Momentum as a measure of goal value best accounts for temporally-extended goal switching". We, however, retained a brief allusion to the physical interpretation of momentum (with a disclaimer) as we wish to highlight the two terms of the momentum product and how they create inertial effects and facilitate persistence in goals.

8. The definition of prospective agent and retrospective agent (p. 9-10) is not clear to me from the main text. There are equations in the Methods, but they are not referenced. Also, the first 3 paragraphs of p.10 are vague. What does it mean that the agent operates "under the assumption of a partially observable Markov decision process"? How is the POMDP defined? What are the states, the observations, the actions etc? How are values estimated? This is crucial, but never shown. Again, we need equations.

   We thank the reviewer for prompting mathematical rigor in these definitions. We elaborated on the definitions of the agents and specifically how its definition falls under a POMDP formulation. We reproduce the excerpt here for specific points raised above.

   We defined the agent as a partially observable Markov decision process, with the current slot configuration as the observed state, selection of cards as actions, and an estimate of the current suit probabilities as the belief state. The agent updates the belief about token-outcome probability of a suit using a delta learning rule,

$$M_g^{(t)} = M_g^{(t-1)} + \alpha(I_g^{(t)} - M_g^{(t-1)}) \tag{11}$$

where $M_g(t)$ is the belief at time t, and $I_g(t)$ is the indicator function of the token outcome (1 if token is received in a round), and α is the learning rate. Using the estimated belief probability, the prospective agent rolls out expectations one step into the future, for two scenarios: receiving a token with the probability M_g, and moving one token closer towards the target ($s_g^{(t)} + 1$), and not receiving the token with the probability $1 - M_g$, and staying in the same slot state ($s_g^{(t)}$). Rolled out future states are discounted by a factor of γ.

$$Q_g\big(s_g^{(t)}\big) = M_g \gamma Q_g\big(s_g^{(t)} + 1\big) + (1 - M_g)\gamma Q_g\big(s_g^{(t)}\big) \tag{12}$$

where $Q_g(t)$ is the value of the goal suit g, $s_g(t)$ is the current slot state of suit g. Specifically $s_g(t)$ is a counter for goal progress, with the above recursive estimation terminating when the current state reaches the target (say 7 tokens).

9. In relation to temporally-extended goal selection / options and evaluation of delayed rewards in a normative framework for decision making, I feel there are at least two concepts that should be included in the discussion.

   a. Time-inconsistent discounting. It has been shown that human decision making is better described by hyperbolic discounting, that is not time-consistent (i.e. the ranking of the discounted values of options is not maintained over time), contrary to exponential discounting. One instance in which this may be an alternative/additional mechanism behind participants preferences, is in describing (at least partially) the tendency of the "retrospective" bias as a function of the progress achieved: if the block switch occurs when we are very close to suit completion, the retrospective option may yield little reward (or reward 1 with low probability), but this would still happen quickly compared to the completion of the prospective suit – and therefore we would still choose the retrospective one. Although this is true also with exponential discounting, I would expect this bias to be stronger with hyperbolic one, in closer correspondence with the results from human experiments reported by the authors.

   This is an excellent point that we had not considered before.

   The prospective agent, as we defined in our manuscript, recursively rolls out estimates of likely future outcomes until termination, steadily discounting future outcomes. Thus, sometimes, it can prefer the suit that is closest to completion as the reward for suit completion need not be discounted by a lot. We originally employed exponential discounting in its formulation. However, we now tested the hyperbolic discounting hypothesis with the prospective agent. We estimated trials-to-completion (delay-to-reward D) for each goal by rolling out outcomes into the future and used the hyperbolic discounting factor ($\gamma = \frac{1}{1+kD}$, $k \in [0, 1]$) to discount the rewards. However, we found no significant difference in behavioral fits in the two variants of the prospective agent with exponential and hyperbolic discounting.

We add the following excerpt to the manuscript.

Time-inconsistent discounting We employed exponential discounting for designing the task-optimized prospective agent. However, given that human behavior is better accounted by hyperbolic discounting [4], we also implemented a prospective agent with hyperbolic discounting. We estimated trials-to-completion (delay-to-reward D) for each goal by rolling out outcomes into the future and used the hyperbolic discounting factor ($\gamma = \frac{1}{1+kD}$, $k \in [0, 1]$) to discount the rewards. However, we found no significant difference in behavioral fits between the variants of the prospective agent with exponential and hyperbolic discounting.

b. Hierarchical reinforcement learning algorithms. Attempts have been made to extend the MDP framework in order to account for temporally-extended options (see "Temporal abstractions through options" in the RL book by Sutton and Barto), by introducing a hierarchy of actions (see, e.g. Botvinik "Hierarchical reinforcement learning and decision making" (2012)). The important difference between the notion of temporally-extended options, vs temporally-extended goals, is that the former implies a "pre-planning" of a sequence of actions (like flipping the same cards for a fixed number of rounds), while the the latter implies re-evaluation of each goal/option at any moment. I think it would be interesting to bring this out.

We have a notion of hierarchy in our algorithm where stay-switch decisions from goals are separate from stay-switch decisions with actions. Thus it is possible to stay with a goal but briefly switch action to explore other options. This ensures continuity in commitment to the goal, while exploring other alternatives to gather information (and potentially trigger a goal switch).

However, the notion of hierarchy where you execute preplanned subroutines / options (and re-evaluation is not done until the end of the option) is not considered in the manuscript and we agree that is an interesting direction for future research. We elaborated on this further by adding the following paragraph to the 'Discussion' section.

There are several limitations of the present work. One is that we introduced several simplifying assumptions into our analysis which when discarded, could open up a realm of other potential algorithmic accounts. For instance, in formulating the prospective agent, we assumed the agent optimizes for immediate rewards delivered upon suit completion. However, it is possible that humans could employ subroutines that are preplanned in line with hierarchical option selection [9], such as committing to a goal for a fixed set of trials. We also observed significant heterogeneity in human goal selection with evidence for the utilization of other strategies (such as TD-persistence) in some individuals. Furthermore, we found evidence that instructions can moderate the influence of momentum computations on goal selection behavior. Thus, the momentum model serves as a coarse descriptor of human goal selection behavior, but not a fully comprehensive account of individual goal selection tendencies.

10. In Fig 3, I feel it would be better to color-code the bars by retrospective/prospective, and group the pairs of bars by token/no-token (so that significant differences would be shown between two adjacent bars, and not spanning between the two groups). Same for Fig 7, 8E, S7 and S8

We revised Figure 3 incorporating the above suggestions. The same was done for the other figures showing switching patterns.

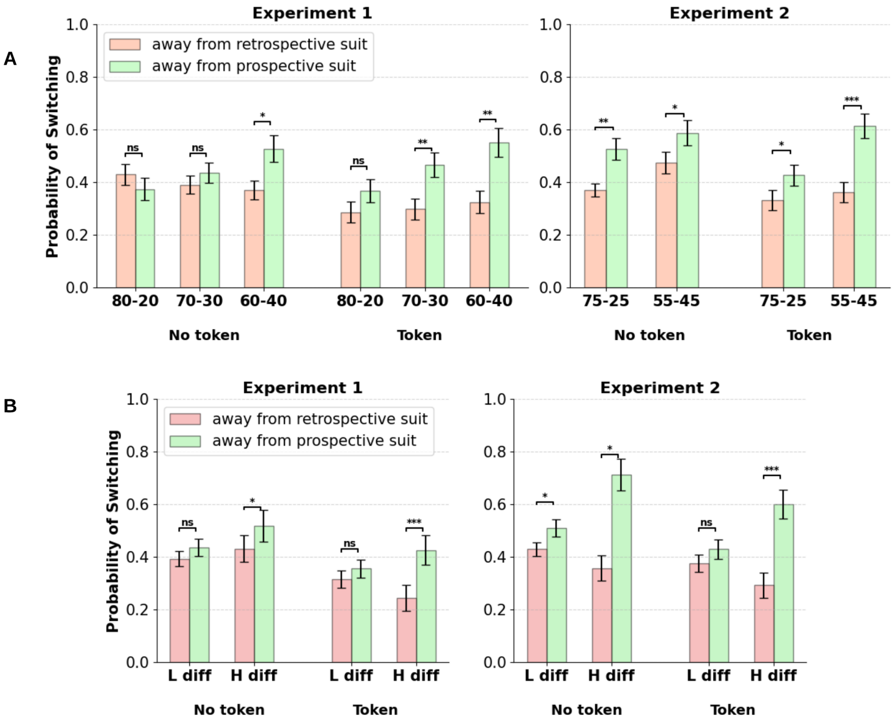

Figure : 3: **A. Participants show greater persistence towards the retrospective suit.** Participants make more switches away from the prospective option than the retrospective option. Switches are split by suit type (prospective/ retrospective), block condition, and whether the round elicits a token or not. Significance markers indicate paired-sample t-tests ($* : p < 0.01, ** : p < 0.001, *** : p < 0.0001$). **B. Switches split by progress differences between the suits.** H diff, L diff correspond to higher and lower progress difference between the suits. Increased persistence with the retrospective suit when progress difference between the suits is high.

11. Fig 6 – and in all model comparison figures. "Score" suggests "the higher the better", but that is not the case for the AIC/BIC scores, that contain a negative log-likelihood – making them into "costs", rather than "scores". Introduce the formulas for the AIC/BIC in the main text. Or clearly indicate to the reader that in Fig.6, "the lower the score, the better the fit". In Fig.6C it is unclear what the quantity being plotted is

We concede that the notion of "score" suggests "the higher the better". However, instead of "costs", we prefer the term "metric", which we now clarify "the lower the metric, the better is the fit" in the manuscript. We also added the formulas for the AIC/BIC metrics.

12. As a general point: all figure legends require more explanation.

    We worked to improve clarity for all figure legends in captions.

13. In Fig.7, I would plot participants and model results in the same graph for easier comparison – e.g. with different shading / dashing

    We realized the figures could be difficult to parse. We reworked the figures for ease of comparisons (employed dashing for models). However, we preferred to place them side-by-side as separate graphs, as we wish to not obfuscate the trends between conditions. We reworked the figure (Figure 8 in the new manuscript) as shown in the document.

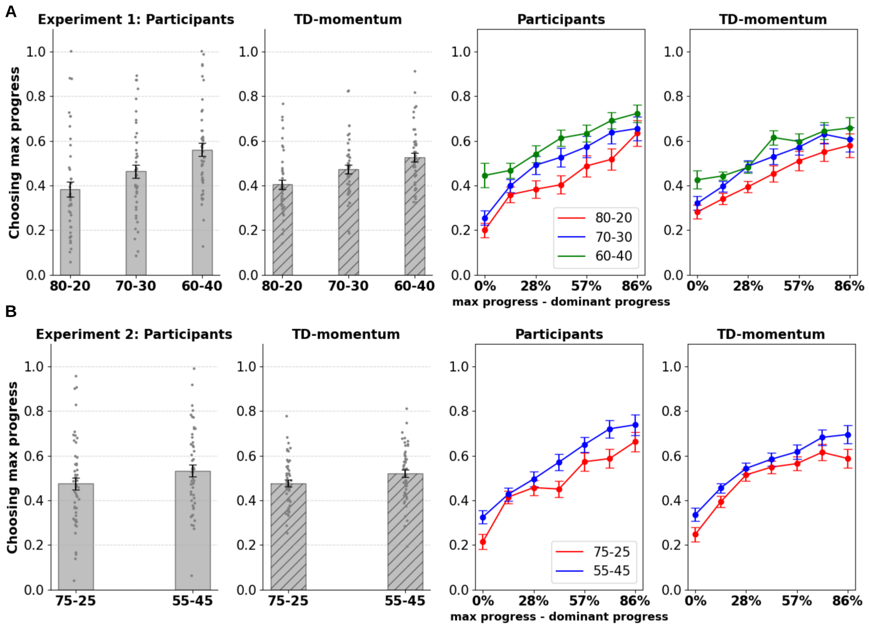

Figure : 8: **Reproducing behavior.** TD-momentum replicates behavioral signatures from the task.

14. In the legend of Fig 4., "Behavior" → "Participants" (the other two are types of agent).

    We corrected this in the manuscript.

15. Fig 4. Labels in captions are wrong – There is no panel C.

    We corrected this in the manuscript.

16. Some minor points about wording

    We made these changes in the manuscript. We, however, retained the 'interaction with block types' as we felt "interaction" is better suited to describe the statistical effect than 'dependence'.

    a. "Relegated to the status of sour grapes" (p.2) – is it a common expression? If not, opt for a more immediate and precise one.

    b. "Lucrative" (p,7) → "advantageous"/"preferable" (?)

    c. "Pales in comparison to" (p.10) → "is much lower than"

    d. "Curtailing exploration" → "reducing" (?)

    e. "Interaction with block types" (p.7) — unnecessary technical stats jargon → "dependence on" (?)

    f. "Interacts with the block condition" (p.5) – same as e.

    g. Remove "clearly" from "clearly evident in the" ecc. (p.11)

Reviewer 2

In this paper, Aenugu and O'Doherty address the issue of goal persistence under the lens of momentum, a concept borrowed from Newtonian physics that is a function of both mass and velocity. In a novel task, the authors observe that people persist in trying to make progress toward the current goal even when switching to a different goal is optimal. By applying the notion that more force is required to change the movement of an object with high momentum, the authors capture human goal persistence behavior across multiple experiments through a newly introduced computational model. The authors also provide a possible explanation for this suboptimal behavior, which might be well-suited (simple and efficient) for more naturalistic Settings.

Goal selection is an important topic that has long been studied but is missing a computational cognitive perspective addressing, testing, and explaining it. I enjoyed reading this article and found the task interesting, the analyses thorough, and the momentum perspective novel and stimulating. However, I have some concerns and clarifications about the modeling approach that the authors should address. In particular, the authors should evaluate and address potential confounding explanations form competing models and artifacts from the experimental design.

Moreover, the authors should take further steps to validate the experimental procedure and their interpretation of the results. In addition, I found the contextualization of the authors' current work and the existing literature insufficient, as it failed to reference many existing, highly relevant published articles. Some clarifications and further information about the methods are also warranted. I list below in more detail the points of major concern that should be addressed to strengthen this submission.

Main questions and comments

I find the authors' work original and their contribution valid. However, I was surprised by how little their study was integrated with the existing literature, including recent and seminal studies.

1. The "retrospective" pull effect the authors find here has been extensively studied by several experiments in humans and other animals, where it was addressed as the "sunk cost fallacy" (Arkes et al., 1985 Organ Behav Hum Decis Process; Sweis et al., 2018, Science; Cheng et al., 2023, Cognition; just to name a few examples). However, the authors never mention this phrase, nor do they cite any relevant literature.

   We referred to "sunk cost" considerations in the original manuscript and also included modeling constraints to account for sunk costs (pgs. 14, 22, 23 in the original manuscript), however, we failed to cite relevant references. We thank the reviewer for bringing this to our attention. We also agree that we should discuss how our task relates to sunk cost mechanisms sooner than was previously done in the manuscript. We now added the following paragraph to the 'Introduction' section of the manuscript.

   Suboptimal human persistence has been extensively documented in prior literature where sunk costs are involved [2] [1], [10]. Humans are known to overpersist in goals that have incurred sunk costs of time, effort, and resources even when it is optimal to abandon them. The sunk cost fallacy demonstrates similar persistence effects in relation to extended goals, the neural basis of which was recently investigated in humans [6]. But not all goal selection decisions we undertake in our daily life occur in the presence of explicit sunk costs. For instance, we can decide to stop working on debugging code and rest instead, with the full knowledge that our work can be resumed later. In this work, we explicitly emphasized investigating human goal persistence patterns in a multitasking setting rather than a sunk cost one. Therefore, we allowed for goals to retain accrued progress having switched away from them. This was done to mitigate the influences of sunk costs in our experiments related to abandoned goals completely losing their value. Consequently the present study deviates from prior work [6] were sunk costs likely exert a potent influence on extended goal pursuit. Moreover, in [6] while overpersistence was observed in behavior in that study, providing a computational account for the overpersistence effect was not a focus. Here, our overall goal is to understand the nature of the overpersistence effect, particularly after the effects of sunk-costs explicitly related to a loss of prior goal progress have been reduced.

   We also referenced a few additional studies in addition to the ones mentioned above that missed our attention previously. We thank the reviewer for prompting us to better contextualize our work in the existing literature.

2. A recent, highly relevant paper presumably escaped the authors' attention. Holton et al. (2024, Nat Human Behav), recently published a study in which they addressed goal persistence bias with a task similar to the authors'. They also provided a computational model of participants' behavior, which they claim is capable of capturing key

behavioral signatures observed in human participants. It is necessary for the authors to:

- Explain how their study differs from Holton et al.'s.

  The main differences between our study and that of Holton are:
  (1) We study extended-goal pursuit in a multitask setting rather than a sunk cost one. We do not reset accumulated progress after a goal has been switched away from which reduces the effect of sunk cost on the behavior as noted in the changes we made to the introductory paragraph noted above.
  (2) Holton et. al introduce a stochastic tree-search model to explain the behavior on their task. This model simulates future trajectories for different options and chooses the option that is forecasted to finish the goal faster. This is essentially similar in spirit to the prospective agent we consider in our task. Moreover, the stochastic tree-search model that is the best fitting model in their study does not account for the over-persistence effect in their task. They, instead, measure the deviance of a participants' behavior from their tree-search model as a measure of their individual persistence bias. In summary, their model does not give an account for why humans tend to overpersist in goals. As a consequence the present study goes beyond the Holton et. al study as providing a framework based on momentum computations for explaining over persistence during goal selection. We discuss this further in the excerpt added to the 'Discussion' section.
  Our study, therefore, goes beyond existing work on human goal commitment by providing a computational basis of overpersistence. [6] observed patterns of overpersistence during extended-goal pursuit in a sunk cost setting. However, their best fitting model was not designed to account for persistence biases displayed by participants in the task. Instead, they quantified an individual persistence bias factor measured as a deviation from their prescribed goal selection model. Our model, in contrast, provides a plausible mechanistic account for participants' persistence patterns during goal-selection. Supporting this claim, the model parameters we fit to individual participants were found to correlate with the extent of individual retrospective preferences manifested in the task. Hence, our momentum model provides a computational basis of human persistence in goal selection.

- State their new contributions clearly.
- Compare their proposed computational model to Holton and colleagues'

  As noted above, the computational basis of over-persistence as a deviance from an optimal model of extended goal pursuit, was not investigated by [6]. The present study addresses this as yet unaddressed question. We propose that momentum computations can result in the tendency to over persist, which could induce persistence in goal selection even in the absence of sunk costs.

3. Moreover, the concept of momentum per se does not imply the presence of a goal in the definition of velocity. The authors thus make an additional step in making an analogy between goal progress and momentum that should be clearly stated and addressed.

   We agree with the reviewer's point. This analogy with physical momentum was previously made in the absence of goals, and our manuscript makes that extension. We highlighted that point in the manuscript.

   The analogy of goal momentum with the physical interpretation of momentum goes only so far as it comprises of two factors, accrued progress (analogous to behavioral mass), and the current the rate of progress (analogous to velocity) that creates resistance to change in valuation. A longer history of reinforcement with an option (accumulated behavioral mass) and a higher current rate of reinforcement both increase momentum and facilitate persistence with an option. This analogy was previously employed in the definition of behavioral momentum [7], where the rate of free operant responding in the presence of a stimulus is likened to the velocity of a moving body, and the resistance to changing the response is likened to its inertial mass. We, further, extend this metaphor to include extended goals, where the momentum of the goal in reaching its desired set point determines its value.

4. Another important issue to address is the fact that it takes participants a few trials to learn the new outcome contingencies. Even in experiment 3, where participants are told what the new probabilities are, they still need to figure out by trial and error which suit has the highest probability of yielding goal progress. Moreover, if participants are not told that consecutive blocks cannot have the same best suit, they might think the current goal suit is the best one even after being informed of a switch. Therefore, some goal persistence should be expected for the few trials immediately following the switch. If that is the case, participants would also fail to explore alternative options – needing to rely on the lack of successful attempts on the retrospective goal rather than experiencing the potential benefits of switching through exploration. One way to address this potential confound would be to introduce forced trials in the experimental design to ensure proper exploration of the alternatives. However, the authors might also propose ways to exclude this alternative explanation through additional analyses of the data they have already collected.

   We performed an additional set of analyses where we investigated participants' choices in the first and second halves of the blocks. We expected that learning effects would prompt an increase in the proportion of times participants chose the dominant suit (one with the highest probability), in the second half of the block. All of the models that we considered for describing participants' choices included a learning component, so we can compare the effects of learning against those of the optimal and fitted models. We included Figure 2D below with the following description at the end of the section "Participants demonstrate a retrospective bias in temporally-extended goal selection".

In summary, the patterns of goal selection in human participants reveal a retrospective bias for accrued progress in the task. Further quantifying the extent of this bias, Figure 2D shows the overall proportion of dominant suit choice in participants contrasted with that of the task-optimized prospective agent. We also split the choices as occurring in the first and the second halves of blocks (averaged over all blocks) to account for any learning effects. Both participants and the prospective agent show learning effects within blocks with the proportion of dominant choice increasing in the latter half of the block. But participants' dominant choice remains significantly lower than that of the prospective agent in both halves of blocks, accounting for suboptimal human performance in the task (elaborated in Figure 4). Lowered learning rates and increased discounting in participants could potentially account for suboptimality in task performance in humans, which we consider to be a potential account for the goal selection patterns seen in humans. This account is tested in later sections by fitting the prospective agent to participants' choices. Furthermore, there are no significant effects of learning or performance shifts across blocks. Supplementary Fig S3 traces the proportion of dominant choice across blocks where we find no significant changes in the selection effects as the task progresses.

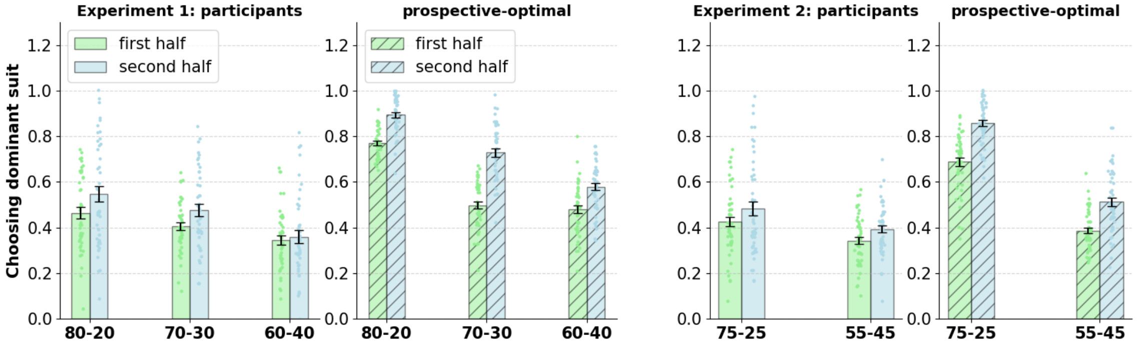

Figure : 2D: **Learning effects within blocks.** Dominant suit choice probability in the first and second halves of the block (15 rounds each). The proportion of dominant choice increases in the second half indicating learning effects within blocks. The choice patterns are contrasted with that of the task-optimized prospective agent indicating suboptimal retrospective bias in goal selection.

From the figure, it is evident that the proportion of dominant suit choice increases in the second half of the block across all conditions. However, the proportion of dominant suit choice in the second half is significantly less than that predicted by a task-optimized prospective agent. Participants chose the dominant option, $> 40\%$ of the rounds in the first half of the block, demonstrating reasonable exploration of the dominant option in the first half of the block, and yet the proportion of dominant suit choice is only marginally higher in the second half, well below that of the optimal prospective agent.

In experiment 3 where we gave explicit instructions to the participants about the current probability context, we explicitly nudged the participants to pay attention to current and alternative goal prospects. This resulted in an increase in dominant suit choice in both the first and second halves of the block, yet still significantly below optimality. We added the below figure (Figure 5C in the manuscript) to the manuscript indicating increase in proportion of dominant suit choice through instructions.

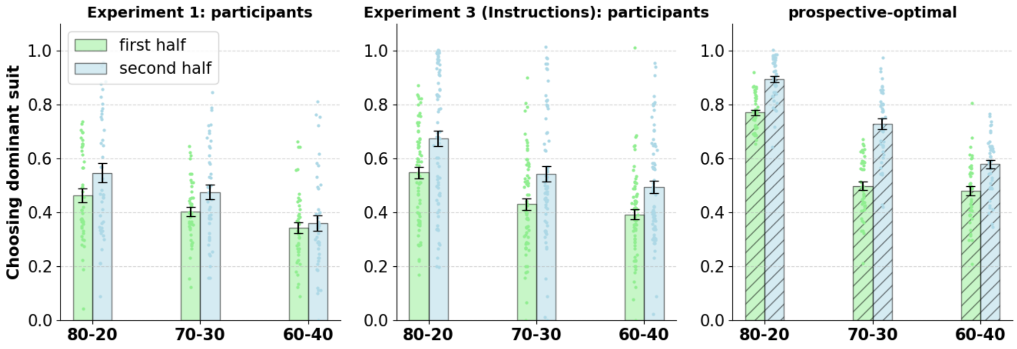

Figure : 5C: **Learning effects within blocks.** Figure shows the dominant suit choice probability in the first and second halves of the block (15 rounds each) in presence and absence of explicit instructions. Preference towards the dominant choice increases in both halves of the blocks indicating a shift towards optimal goal selection in the presence of instructions.

Moreover, TD-momentum fit to participants' choices can replicate learning effects seen in participants. We added the below figure (supplementary figure S9) comparing participants' learning effects with those of model-predicted effects.

5. I have some additional concerns regarding the proposed computational model. It is unclear to me which γ was used for the prospective (optimal) model (p. 10). With high levels of delay discounting, might we expect the optimal model to perform more similarly to the participants?

   We added the following excerpt giving more details about the γ factor from the task-optimized model.

   We optimized the parameters of the prospective agent (parameters and action selection process elaborated in the Computational modeling section of Methods) for task performance (total number of suits completed). Optimal discount factor for this agent is γ is 0.95 for experiment 1 and 0.93 for experiment 2. The prospective agent, thus optimized for the task, outperforms human participants significantly (see Figure 4; performance distinctions elaborated in a later section).

   The discount factor of the prospective agent fit to participants' choices is 0.7 in experiment 1 and 0.6 in experiment 2. The prospective agent when fit to participants' choices reproduces participants' choice patterns to some extent (though not as well as

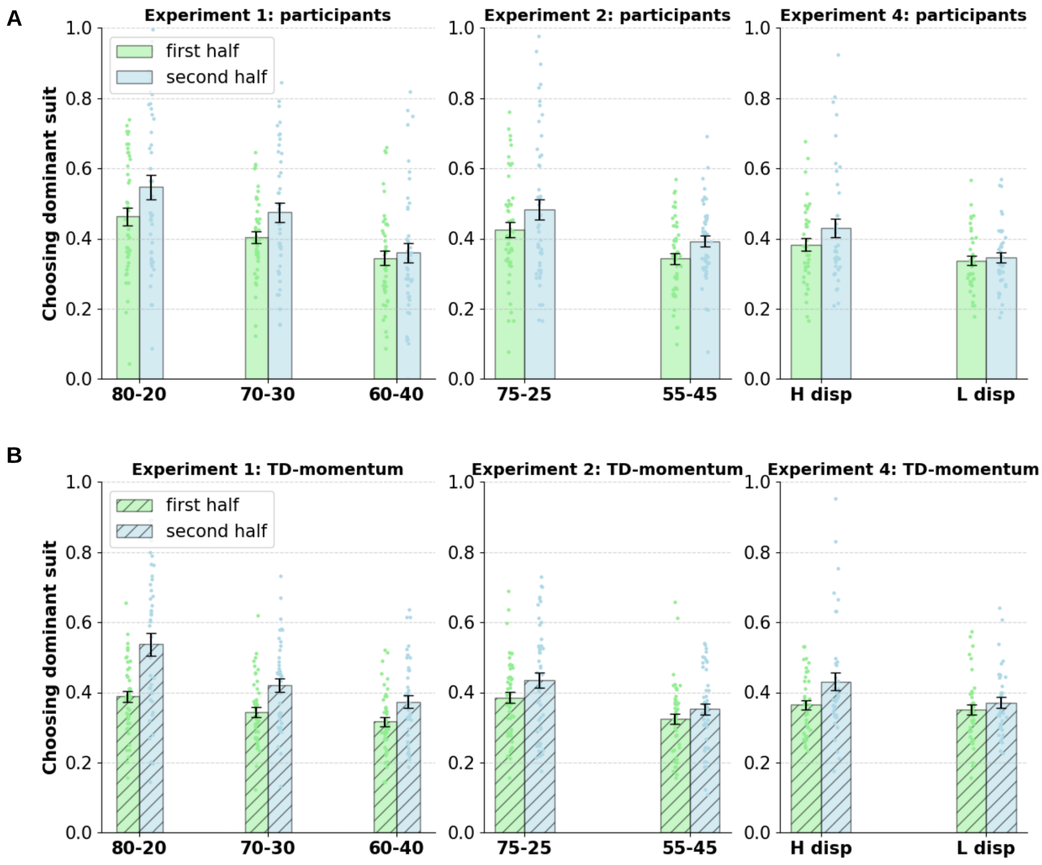

Figure : Fig S9: **Learning effects within blocks.** Figure shows the participants' dominant suit choice probability in the first and second halves of the block across all experiments replicated by TD-momentum.

TD-momentum). This finding prompted us to perform experiment 4 to better dissociate the prospective and momentum predictions, where we show that TD momentum explains behavior substantively better than the (fitted) prospective agent, even though as noted below, TD momentum does not capture human behavior perfectly in that experiment.

6. The authors do not justify the presence of a bias term for each goal in their TD-momentum model (p. 13). More importantly, it is unclear why the bias would need to be updated via the learning rule. Is this mechanism necessary for the model to capture participants' behavior? If so, it would be useful to show this necessity in the supplements (e.g., comparing to a simpler model lacking this term). Moreover, the authors should explain how this term affects their interpretation of momentum and the way it is applied to human goal persistence.

   This is an essential point that we missed highlighting in the manuscript. We now added the following excerpt to describe the necessity of the goal-specific bias term.

   The goal-specific bias term (b_g) is included to ensure that the goal value is updated even when the progress is at $m = 0$. When one chooses the goal when its progress is zero and repeatedly fails to achieve outcome, its value is depreciated through the bias term. The bias term also ensures goal value is retained when the goal target is accomplished (upon which the progress m resets to zero), and can increase the chance of choosing the same goal again even though the value component from the term $v_g m$ is zero.

7. It is unclear to me how the authors modeled goal selection. If they used information from goal probes, was the assumption that goals remained the same before/after the next goal probe? If they inferred it from participants' choices, how could the model (and associated likelihood) distinguish between an unlikely, yet possible, goal-incongruent action vs. a switch in goal?

   This is another key missing element from the methodology. We thank the reviewer for bringing this to our attention. We included the following excerpt in the "Model fitting procedure" in Methods.

   We fit both participant card selections and goal probes in all models except for the Rescorla-Wagner model where we just fit participants' choices (where there is no notion of an extended goal).

   Goal probe likelihood is estimated only in the rounds where goal probes are prompted in the task. In the rest of the rounds, goals are chosen by the model and action probabilities are estimated according to the goal specified by the model and thus facilitating the estimation of participants' likelihood of action (card) selection in all rounds. Model goals are aligned to the participant goals in the rounds with the goal probes, so the model can compute stay/ switch decisions from the participant-aligned goals following the goal-probe round.

Our goal-action selection mechanism is hierarchical with dissociation between goal and action selection processes (elaborated further in Algorithm 1 below). Therefore, we allow for actions to diverge from goals, so it is possible that a participant stays on the higher level goal and yet can switch actions occasionally (resulting goal-incongruent choices). We expect these goal-incongruent choices to be fewer in number but not zero (to account for exploration). In the rounds where we did not probe the participant's goal, we rely on the model to provide an estimate for the goal. If the action is incongruent to the goal, the likelihood for the goal-incongruent action can be high or low depending on the softmax temperature parameter set for exploration. We elaborated more on the statistics of goal-action congruence in participants in the comments below.

We also included the following pseudocode for the goal selection process for further clarity:

Algorithm 1 Algorithm for goal selection

Q_g: goal value where $g \in \{\text{cat, hat, car}\}$
β_g, β_a: softmax temperatures for goal and action selection
c_g: goal preservation/ choice kernel
c: cost of switching between goals
G: current goal (none at start)
for block in (1, *num_blocks*) **do**
    Initialize card probabilities
    for round in (1, *num_rounds*) **do**
        Get goal values ($Q_{cat}, Q_{hat}, Q_{car}$) *// Using TD-momentum/ prospective*
        Calculate advantage of current goal G:
        $A_G = Q_G - \max_{g \neq G} Q_g$
        Choose to stay with/ switch from the current goal:
        $p_{\text{stay};G} = \frac{1}{1+\exp(-c-c_G-\beta_g A_G)}$ *// Choose goal*
        In case of a switch, chosen the new goal according to goal values
        Update current goal G
        Choose to act according to the current goal or explore
        $p_{\text{stay};G,A} = \frac{1}{1+\exp(-c-c_G-\beta_a A_G)}$ *// Choose action*
        Observe outcome
        Update goal probabilities
        Update goal values
    end for
end for

8. Simulated behavior from any of the models the authors present does not match the participants' behavior well in Experiment 4. The authors recognize this limitation but do not address it. Could the authors explain the mismatch, and propose extensions

of their model to address it? Before diving into plots and analyses of interests, some task validation steps should be performed

We also highlighted the following points in the manuscript, noting deviations of TD-momentum from the participants. We also propose potential extensions to the model (say including specific inductive biases).

- Moreover, goal selections are uniform with slight biases towards specific suits on account of them having variable length targets. For instance, participants selected the hat suit significantly lower than the *cat* or *car* suits in the low disparity condition, perhaps accounted by preferences to the options at extremes (in terms of target length)
- The TD-momentum model did not fully capture the specific bias against the *hat* suit displayed in the *L disp* condition, nevertheless its distribution of goal selections appear to be closer to the pattern found in human participants' choices. Finer deviations in goal selection patterns between the TD-momentum model and human behavior can potentially be attributed to idiosyncratic goal selection patterns in humans that are not factored into the model. The deviations could also arise from systematic biases (such as a preference for options at the extremes), which could be included in the model as inductive bias components.

Regarding task validation steps, the primary task manipulation is same as the previous experiments. We introduced block shifts with dominant suit switching between adjacent blocks. We also introduced high disparity and low disparity blocks, varying the difference between the dominant and inferior suits. We expected participants to be sensitive to both these manipulations like we observed in the previous experiments. The excerpt indicating that participants are responsive to these manipulations is reproduced below.

Figure 9B shows participants' goal selection probabilities split by the dominant suit condition. Participants' selected the *cat* suit with higher chance in the *cat*-dominant blocks, and so forth for the other suits, indicating sensitivity to token contingencies. Participants also displayed sensitivity to relative distinctions between the suits with a lowered proportion of dominant suit choice in the low disparity blocks compared to the high disparity blocks (Figure 9C).

9. The authors seem to rely a lot on goal probes. Therefore, it is necessary to understand how consistent responses to probes were with participants' responses in the task. It also seems important to address whether/how goal probes might have impacted the participants' choices throughout the task. For instance, the probes might have prompted goal re-evaluation. Alternatively, goal probes might have prompted participants to act consistently with their declarations beyond what they thought was optimal for the task (e.g. to avoid cognitive dissonance), yielding goal persistence effects to suboptimal levels. Were goal switches more/less frequent around goal probes?

We agree with the reviewer that the analysis on the consistency of the goal probes needs to be included in the manuscript. We included the following excerpt in the Methods followed by plots in the supplementary.

Goal probes

We elicited goal probes from the participant every third round in the task. We relied on goal probe estimates to account for model predictions of goal persistence that is different from action selection. To ensure reliability in goal probes, we measured goal-action congruence (whether the participant made an action that is congruent to the goal in the round following the goal probe prompt. Supplementary Fig S2.A shows goal-action congruence density histograms plotted across all experiments. Mean congruence in goal probes are 80%, 78%, 76%, and 73% for experiments 1 to 4.

We also estimated the proportion of the total number of switches that the participant made in the task that followed a goal probe. This is to verify whether the goal probe prompt itself influenced subsequent goal selection (either prompting a switch / prompting persistence in the specified probe). Figure S2.B shows the mean proportion of switches are 31%, 32%, 29%, 32% for experiments 1 to 4. As the goal is probed every third round (in 33% of the rounds), the proportion of switches around the goal probes are in line with the chance expectation of 33%.

Goal-action congruence is high, yet not perfect in participants. We anticipated this mismatch by our hierarchical assumption in our modeling, by dissociating participant goal and action selection. Participants can value a goal above other alternatives, yet can temporarily explore other options before revisiting it. We found such patterns of revisiting in participants (Fig 3), indicating a persistence in goal valuation. However, we still expect noise in goal probes in a portion of less-engaged participants (perhaps those showing low goal-action congruence) and that is a fundamental limitation in probing intrinsic goal states in participants. This noise could account for the models not perfectly capturing goal selection patterns in the task.

10. Since switches occur at regular intervals, people might learn the structure of the task over time. Was persistence less strong at the end, compared to the beginning of the Task?

    We examined how the proportion of dominant choice varies as the task progresses. We split the blocks into groups of 3 and tracked the mean proportion of dominant suit choice with task progress in Figure S3. We see only dominant choice proportion marginally decrease in the last 3 blocks compared to the first 3 blocks in experiment 2, indicating only minor differences in persistence levels as the task progressed. We included this figure in the supplementary (reproduced below).

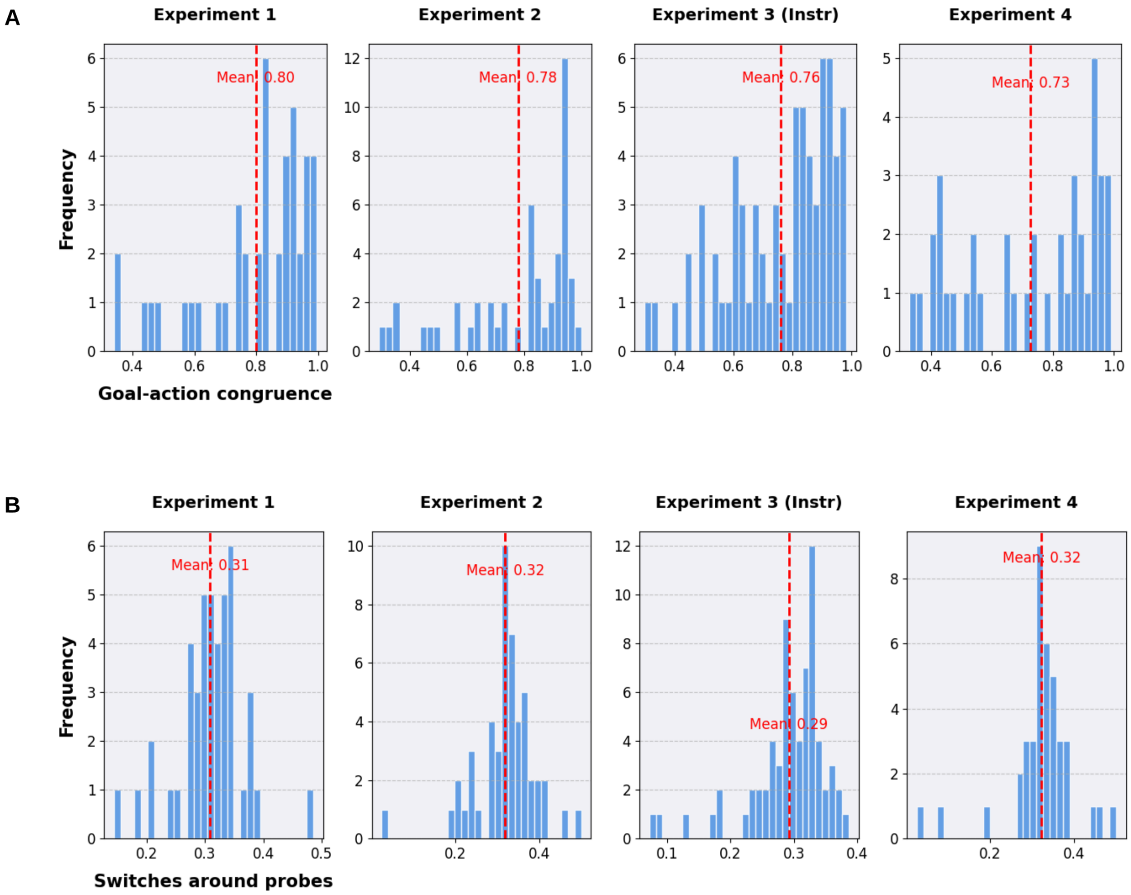

Figure : S2: **A. Goal-action congruence** Figure shows the proportion of rounds where the action is performed in congruence in with the goal probe in the next round. **A. Switches after goal probes** Proportion of the total number of switches made by participants that occur in the round following the goal probe.

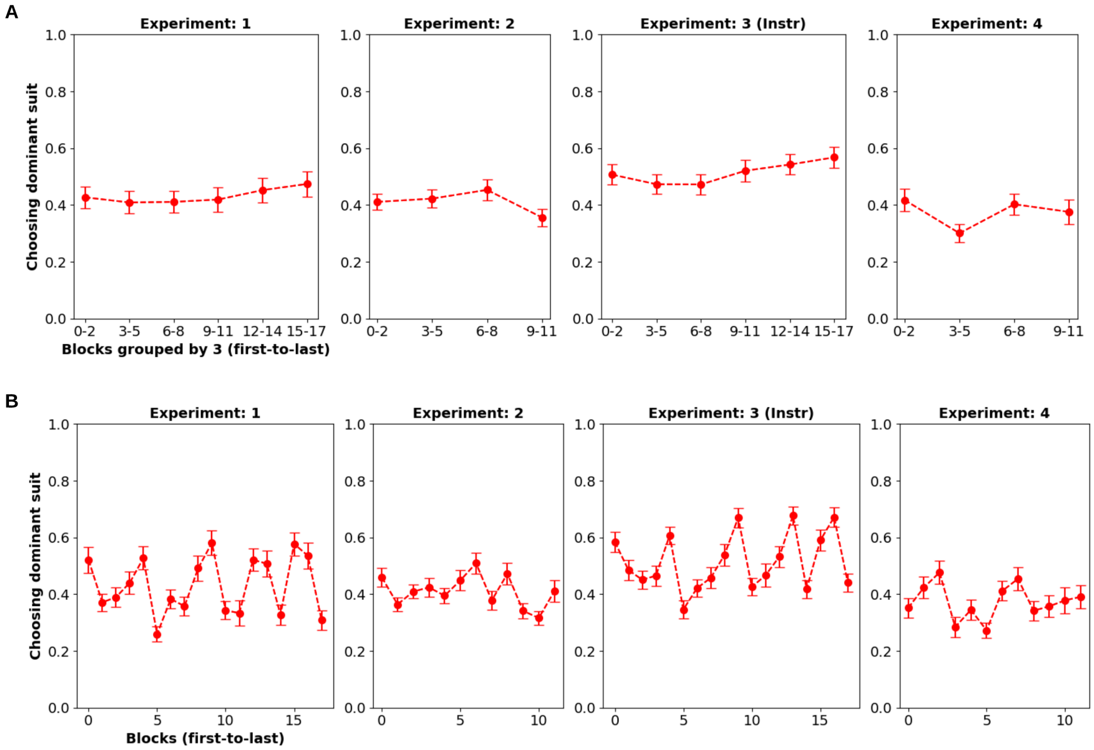

Figure : S3: **A. Proportion of dominant choice traced with task progress** Blocks are split into groups of 3 for tracking progress through the task. There is no indication of increased persistence at the end of the task (last three blocks) compared to the beginning (first three blocks) except in experiment 2 where there is a marginal decrease in preference towards the dominant suit at the end. **B.** Proportion of dominant suit choice with no grouping of blocks. Oscillations in dominant choice proportion corresponds to the encountered block condition.

11. The authors also state that "It is more lucrative to switch away from the retrospective suit in high disparity blocks than in low disparity blocks" (p. 7). However, this is not always the case: if the horizon is long enough, it should always be optimal to switch. By contrast, if only a few trials remained in the task, it might be more advantageous to stick to the current goal.

    We agree that the task horizon would effect choices. We plotted the dominant suit choice as the task progresses in the supplementary figure S3 (reproduced below).

    - Were participants aware of how many trials were left in the trial/block?

      Participants are aware of the number of blocks left in the task. We prompted the beginning of each block with a counter, say "Block 7/ 18". However rounds (trials) were counted without an upper limit, say "Round 28" instead of "Round 28/ 30" (an omission on our part while designing the task). However, during the instructions, we informed the participants that each blocks consists of 30 rounds (trials). We added this information in the methods.

    - Did their behavior change towards the end of the task?

      Supplementary Fig S3.B shows the block-wise dominant suit choice as the task progresses. The dominant suit choice proportions oscillates as per the block condition of a specific block (say 80-20/ 60-40). We did not find any significant changes in behavior towards the end of the task.

12. The last section on simulations is useful to test whether the observed suboptimal behavior corresponded to optimal choices in a different, and more naturalistic setting. However, the authors should address (at least in writing) other possible explanations. For instance, Cheng et al. (2023, Cognition) find that humans are biased towards keeping their goals consistent as it helps inform others of one's intentions in social contexts. Therefore, people might be biased toward goal persistence even in asocial settings out of an acquired habit. The Discussion section failed to mention any existing limitations of the current work, and how they might be addressed by future work.

    We added the following excerpt to the "Algorithmic complexity and normative account of momentum" to expand on other normative accounts of goal persistence.

    Momentum computations can be efficient, yet in scenarios with sudden upheavals of fortunes, as is common in quotidian experiences, they can lead to suboptimal decisions. Also, momentum computations and concomitant goal persistence can have adaptive features when there is uncertainty in the environment. Even when an option is devalued, one might be uncertain when it might be valued again, or whether viability of alternative options would remain stable enough to warrant a switch. Furthermore, persistence in goals can have other advantages in social settings, such as stable inference of one's goals by peers [5], [3], all the while avoiding the inefficiency of incurring cognitive costs of goal switching.

We also added the following paragraph in the discussion to discuss the limitations and extensions of our current work.

There are several limitations of the present work. One is that we introduced several simplifying assumptions into our analysis which when discarded, could open up a realm of other potential algorithmic accounts. For instance, in formulating the prospective agent, we assumed the agent optimizes for immediate rewards delivered upon suit completion. However, it is possible that humans could employ subroutines that are preplanned in line with hierarchical option selection [9], such as committing to a goal for a fixed set of trials. We also observed significant heterogeneity in human goal selection with evidence for the utilization of other strategies (such as TD-persistence) in some individuals. Furthermore, we found evidence that instructions can moderate the influence of momentum computations on goal selection behavior. Thus, the momentum model serves as a coarse descriptor of human goal selection behavior, but not a fully comprehensive account of individual goal selection tendencies.

13. Additional information about the analyses and methods is also needed.

    - The authors perform analyses split by high vs. low progress differences between the prospective and retrospective suits. However, I could not find information about the cutoff between high and low differences, making it hard to interpret related results.

      We added the following clarification to the manuscript.

      Retrospective switch propensities are also sensitive to the relative progress differences between the two suits and are lower when the difference is high. Figure 3B shows switching proportions away from prospective and retrospective suits split by high ($> 50\%$) and low ($\leq 50\%$) progress difference between the two suits.

    - In the Methods section (p. 25), variables need to be explicitly defined. For instance, Ig only gets defined later on, and Mg never.

      We moved the description of the algorithm to the Methods section, where we corrected this omission.

      The agent updates the belief about token-outcome probability of a suit using a delta learning rule,

      $$M_g^{(t)} = M_g^{(t-1)} + \alpha(I_g^{(t)} - M_g^{(t-1)}) \tag{13}$$

      where $M_g(t)$ is the belief at time t, and $I_g(t)$ is the indicator function of the token outcome (1 if token is received in a round), and α is the learning rate.

    - I could not find information about the participants' mean age and SD. Given the wide age range presented in Table 1, the authors might also want to address potential age effects in their results.

Table 1: Participant recruitment

	Participants	Median time (mins)	Age range (mean ± std)	Female/ Male
Experiment 1	44	31	18-64 (28.6 ± 9.5)	13/ 31
Experiment 2	49	24	20-68 (29.2 ± 8.8)	20/ 29
Experiment 3	67	33	19-52 (28.1 ± 7.6)	32/ 35
Experiment 4	39	26	19-57 (31.1 ± 10.0)	18/ 21

We updated the table to include mean and SD of participants.

Interestingly, we have not found any significant age effects in our results. We analyzed retrospective preferences, goal/ action switching rates, and model-predicted γ and verified if it correlated with age. We found no significant correlations with age.

- Lastly, it would be useful to have an upfront overview of the four experiments and the simulations stated before diving into the main Results section.

  We added the following excerpt to the task description section in Results.

  We include four experiments using this paradigm. Experiments 1-3 have uniform targets for all suits, with each suit comprising of seven tokens of one kind. Experiment 1 has 18 blocks with 30 rounds each, with three different probability structures: 80-20, 70-30, 60-40. In the 80-20 block (say), the dominant suit is available with 80% chance and the two inferior tokens are available with 20% chance. Experiment 2 has 12 blocks (30 rounds each), with two different block types: 75-25 and 55-45. In both experiments 1 and 2, our instructions are limited in that, within each block, one specific suit has the highest probability, and the probabilities change with each block switch. In experiment 3 (which is a repeat of experiment 1), we gave participants more information about their current block context. Before each block began, we specifically informed them that they are in an 80-20 context, without telling them which specific suit is available with 80% chance (more details on task design and instructions are elaborated in the Methods section). In experiment 4, we employed non-uniform targets for different suits and explored its impact on validating our computational hypothesis.

14. Minor corrections

    More details and clarifications are needed in the task description in the Results section.

    - The number of blocks and trials per block appear in the Methods, but would be helpful to have in the results, too.

      We added this detail to the results section.

    - The phrase “to ensure the goal selection is not tagged to stimuli” is unclear, so the design choice does not appear well-justified (p. 3).

We elaborated on this more by adding the following excerpt.

Throughout the game, cards serve to stand-in for the suits, and to collect tokens for a specific suit, participants need to flip the cards associated with the suit. Through this design, we aimed to eliminate stimulus-related value mapping, which would likely emerge if we were to directly display the suit stimuli (say images of *cat*, *hat*, *car*), and elicit the construct of goals in participants. If say, a participant persists in the goal of pursuing the *cat* suit, they would pick the relevant cards (the *basket* card) when it is available, and the *mat* card when it is not). By making the associated cards appear at random, we mitigated stimulus-elicited persistence effects in our design.

- My understanding is that switching from one suit to another does not forgo previously accrued goal progress. If this is correct, it would be helpful if the author state this explicitly (instead/in addition to declaring "no penalty").

  We added the below clarification in the task description in the results section

  Participants are free to select any of the suits at any time and can switch away from it at will without losing progress accrued toward the suit's target.

- The authors should clarify how a block transition is signaled.

  We added the below clarification in the task description in the methods section

  For experiments 1 and 2, we instructed the participants that token probabilities are constant within a block but shifts across blocks. We included a quiz during instructions to ensure they understand block switches and differing token probabilities. In the experiment, block transitions are signaled with a screen that displayed the text ”This is the beginning of a new block" for 2 seconds.

- The phrase "Participants can select between [among] three suits in the task" might mislead a first-time reader into thinking an explicit goal selection is made (p. 5).

  This phrase is no longer in the revised section

- Clarify (here, and in the Methods) exactly which blocks are high vs. low disparity, instead of giving examples (p. 5).

  We added the following excerpt to the task description in the results section.

  Furthermore, we designed blocks in the game so that there is a dominant suit in each block (counterbalanced across all suit types), such that flipping its cards gives tokens with the highest probability (in Figure 1D, the *cat* suit is the dominant one with 0.8 probability in block N). The other two suits have identically inferior token probabilities (both *hat* and *car* with 0.2 probability). Pursuing the dominant suit type on each block gives participants the best chance at success in suit completion. We also varied the probability disparity between the dominant and the inferior suits to see how it modulates goal selection. We expected that the task pressure to select the dominant suit would be higher in the high disparity blocks (where probability difference between the dominant and the

inferior suits is high) and would be reflected in participants' goal selection patterns. We instructed the participants of this task manipulation, explaining that within each block there is a specific suit which has the highest chance of token collection. However, we did not emphasize the design manipulation that the dominant token in one block becomes an inferior token in the adjacent block.

15. Some corrections need to be applied to figures and/or figure captions.

    - In Figure 4, subplot B is mislabelled and a reference to subplot C is missing.
      We corrected this in the manuscript.
    - Consider adding an explicit reference to experiment 3 in Figure 5 or its caption.
      We made this change to the figure.
    - The "Prospective" label is somewhat confusing, as it is unclear whether it refers to the optimal model or the Prospective model fit to human participants (p. 11).
      We added a clarification that it is the task-optimized prospective model
    - I also have a suggestion for an edit to the Discussion section. On p. 22, the authors state that they informed participants of "the token-outcome contingencies of the current block". This can easily be interpreted as telling participants about which suit was the optimal one in a given block, which is incorrect.

    We paraphrased the above statement as reproduced below.

    We conducted a variant of the task giving explicit instructions on the contextual prospects of different goals. By alerting the participants about the probability structure within a block, we expected the inference of the identity of the dominant suit would be relatively easier and more salient.

16. Additional minor corrections and typos are listed below.

    We thank the reviewer for pointing these out. We corrected these points in the manuscript.

    - Should citations be compressed? E.g., [2-4] instead of [2],[3],[4]
    - Sometimes, the suit name is capitalized in the text, but should instead be lowercase and in italics for consistency (e.g., p. 4, "HAT"). Similarly, "Cat" should be lowercased on p. 18.
    - Replace"Participants overall, " with "Overall, participants " (p. 5).
    - No comma needed after "closer one to the target" (p. 7).
    - Insert "The" before "Persistence in goal selection that we describe here" (p. 8).
    - In describing the momentum model on p. 14, the authors state "if there is a unit of progress $(m \to m+0.14)$" etc. However, the correspondence between a unit of progress and 0.14 is not true for experiment 4, so the authors might want to provide a more general description.

- Replace “whether TD-momentum provide” with “whether TD-momentum provided” (p. 15).
- Insert “The” before “Dominant token” (p. 24).
- Insert “the” before “state of the goal” (p. 26). “Rescorla” and “Wagner“ should be capitalized in “rescorla-wagner” models.

References

[1] Hal R. Arkes and Peter Ayton. The sunk cost and concorde effects: Are humans less rational than lower animals? *Psychological Bulletin*, 125:591–600, 1999.

[2] Hal R Arkes and Catherine Blumer. The psychology of sunk cost. *BEHAVIOR AND HUMAN DECISION PROCESSES*, 35:124–140, 1985.

[3] Shaozhe Cheng, Minglu Zhao, Ning Tang, Yang Zhao, Jifan Zhou, Mowei Shen, and Tao Gao. Intention beyond desire: Spontaneous intentional commitment regulates conflicting desires. *Cognition*, 238:105513, 9 2023.

[4] Shane Frederick, George Loewenstein, and Ted O’Donoghue. Time discounting and time preference: A critical review. *Journal of Economic Literature*, 40:351–401, 1 2002.

[5] Erving Goffman. The presentation of self in everyday life. *Social Theory Re-Wired*, pages 450–459, 6 2023.

[6] Eleanor Holton, Jan Grohn, Harry Ward, Sanjay G. Manohar, Jill X. O’Reilly, and Nils Kolling. Goal commitment is supported by vmpfc through selective attention. *Nature Human Behaviour 2024 8:7*, 8:1351–1365, 4 2024.

[7] John A. Nevin, Charlotte Mandell, and Jean R. Atak. The analysis of behavioral momentum. *Journal of the Experimental Analysis of Behavior*, 39:49–59, 1 1983.

[8] Alex Pine, Ben Seymour, Jonathan P. Roiser, Peter Bossaerts, Karl J. Friston, H. Valerie Curran, and Raymond J. Dolan. Encoding of marginal utility across time in the human brain. *Journal of Neuroscience*, 29:9575–9581, 7 2009.

[9] Richard S. Sutton, Doina Precup, and Satinder Singh. Between mdps and semi-mdps: A framework for temporal abstraction in reinforcement learning. *Artificial Intelligence*, 112:181–211, 8 1999.

[10] Brian M. Sweis, Samantha V. Abram, Brandy J. Schmidt, Kelsey D. Seeland, Angus W. MacDonald, Mark J. Thomas, and A. David Redish. Sensitivity to “sunk costs” in mice, rats, and humans. *Science*, 361:178–181, 7 2018.

[11] John N Tsitsiklis and Benjamin Van Roy. Analysis of temporal-diffference learning with function approximation. *Advances in Neural Information Processing Systems*, 9, 1996.
