# Supplementary material for: Building momentum: A computational account of persistence toward long-term goals
Source: PLoS Comput Biol. 2025 May 14;21(5):e1013054. doi: 10.1371/journal.pcbi.1013054 (PMC12101773; doi:10.1371/journal.pcbi.1013054)
Supplement: Attachment — Submitted filename: PlosCB_reviewers_revision_2.pdf [file pcbi.1013054.s020.pdf]

Address to reviewers:
Building momentum: A computational model of persistence toward long-term goals

Sneha Aenugu, John O'Doherty

April 2025

Below, we address the reviewers' outstanding comments point-by-point. Reviewers' comments are indicated in black, our comments addressing the reviewers are in red, and the changes reflected in the manuscript are indicated in blue.

Reviewer 1

I would like to compliment and thank the authors for taking great care in addressing most of the questions from both us reviewers.

However, I feel that the task contingencies are still not explained clearly, which has been a source of a major misunderstanding on my side – about which I had an exchange with the editor. My original question –probably unclearly formulated– was about whether or not the *card* chosen by the participant had any effect on the performance.

What I meant by *card*, confusingly, was the *token represented by the card* (car, hat, cat), and not the specific picture on the card (mat, wardrobe, key, etc).

For instance, in Fig 1, in the first block of Exp 1 one has
$p(car) = 0.8$
$p(cat) = 0.2$
$p(hat) = 0.2$
According to my understanding, this meant that whichever card (the actual card, with the token-related symbol on it) the participant picked, the probability of obtaining a token (say, "car") would always be the same (0.8 for the "car").

Instead, as I am aware the editor confirmed with you, these probabilities should be expressed more rigorously as *conditional probabilities* as follows:
$p(car\ token|car\ related\ card) = 0.8$
$p(car\ token|cat\ related\ card) = 0$
$p(car\ token|mat\ related\ card) = 0$
$p(cat\ token|car\ related\ card) = 0$
$p(cat\ token|cat\ related\ card) = 0.2$
$p(cat\ token|mat\ related\ card) = 0$
$p(mat\ token|car\ related\ card) = 0$
$p(mat\ token|cat\ related\ card) = 0$

$p(mat\ token|mat\ related\ card) = 0.2$

In the new text, we read: "Participants receive tokens upon flipping cards with a given probability. If they receive the token for a suit type in a round, it goes into its designated slots and the participant proceeds to play another round."

A whole paragraph later (!), we have a description of the probabilities: "Furthermore, we designed blocks in the game so that there is a dominant suit in each block (counterbalanced across all suit types), such that flipping its cards gives tokens with the highest probability (in Figure 1D, the cat suit is the dominant one with 0.8 probability in block N). The other two suits have identically inferior token probabilities (both hat and car with 0.2 probability)."

It currently takes 5 (!) paragraphs to get to the to these probabilities. They should come immediately (right after "Participants receive tokens upon flipping cards with a given probability") with the help of equations, and also with a clear graphical representation of the *conditional* probabilities in Fig. 1.

This points back to one of the first comment I made in the first round of revisions: the exposition should be more succinct and rely more on mathematical formulas.

I expect the readers of PLOS Computational Biology to be generally well versed in math, and this should not be an obstacle; rather, I feel it would help most readers go through the setup and the results. Currently, I feel that the exposition is too verbose, at the cost of readability.

For this reason, I would strongly recommend to make further edits in this direction, to make justice to an otherwise interesting and solid work.

We thank the reviewer for their patience dealing with the suboptimal task description. In addition, we thank the editor for facilitating subsequent clarifications and also for the recommendations on how to improve our narrative. We incorporated the recommendations and also reduced the verbosity. We added a succinct description of the task and our experimental manipulations in the main results section and elaborated with additional details in the methods section. We also revised the task figure to make the experiment more intuitive and added a graphical representation of the probabilities in the task.

Also, we made sure we provide suitable mathematical descriptions of all the algorithms described in the paper, including the model-fitting process.

Here is the updated description of the task contingencies:

Results

Suits task: a paradigm for studying extended goal pursuit

We introduce a suit collection paradigm to simulate extended goal pursuit inspired by traditional card games. A suit in the game comprises a specific number (say seven) of tokens of the same kind. The objective for the participants is to collect as many suits as they can and win points in the game (which ultimately translates to a monetary bonus). There are three different types of suits available (*cat*, *hat*, *car*) and participants can freely switch between collecting different suits without penalty to earn points in the game. Participants only receive points when a suit is completed without any partial credit for any incomplete suits.

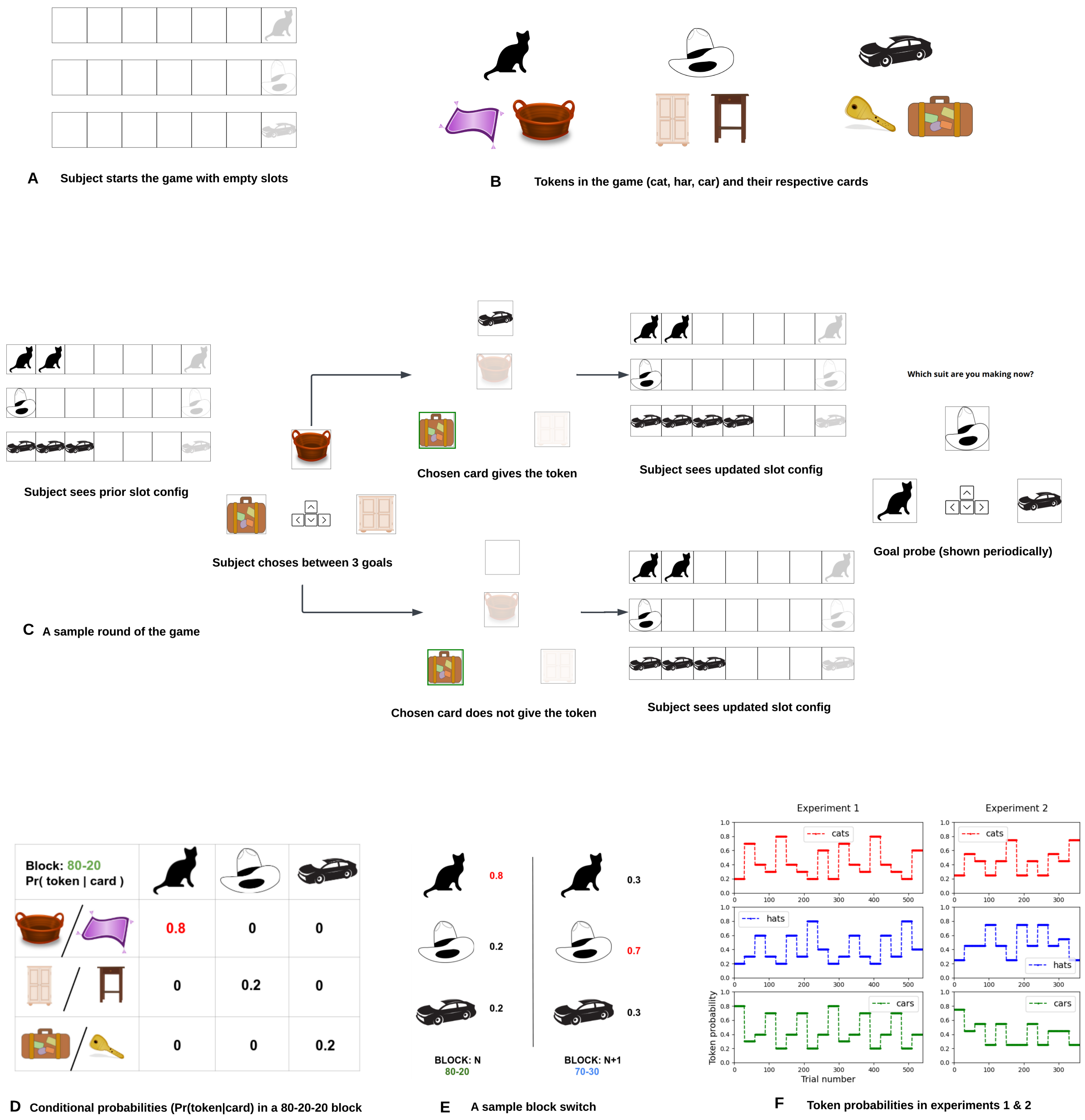

Figure : **Suits task.A.** Participants start with empty slots to collect tokens for each of the three available suits (*cat, hat, car*). **B.** Each suit type is mapped to two distinct cards which serve to stand in for the suits in the game. **C.** Panel shows a sample round of the task: 1. Participants see suit collection progress of all three goals. 2. Participants pick a card corresponding to one of the three goals. 3. Participants sees the outcome of their action 4. If a token is received, slots are updated. 5. Periodically, participants report the suit they are pursuing. **D. Conditional probabilities**. $\Pr(token|card)$ for different token-card combinations are shown for the 80-20 block. Selecting the *basket* or the *mat* card gives you the *cat* token with a high probability of 0.8, making *cat* the dominant suit in the block. **E. Block shifts** Each block has fixed probabilities with one dominant suit and two inferior suits of identical probabilities. The dominant suit is switched across adjacent blocks (*Cat* is most abundant in block N while *hat* is the most abundant in the next one). **F. Token probabilities.** Two experimental variants (experiment 1 and 2) with probability configurations specified for each suit. **Figure credits.** We used images from https://openclipart.org/ to generate the figures. See the Task design section in Methods for full image credits.

Gameplay

The game is played in blocks with each block having 30 rounds. A sample round of the gameplay is shown in Figure 1. Participants start with an empty set of slots (7 for each token type as shown in Figure 1A).

State: The game state is indicated by the current slot configuration which indicates progress made toward different suits. An example slot configuration occurring a few rounds into the game is shown in Figure 1C.

Actions: There are 6 cards in the game, each corresponding to a specific suit type (two cards are allotted for each suit, see Figure 1B). On each round, a participant is shown three cards (one card for each suit). Participants can choose to flip between one of the three cards shown to receive tokens for a suit (see Figure 1C). For example, if a participant wishes to acquire a *car* token to collect the *car* suit, they would pick the *key* or the *baggage* card (whichever is available in the round) to try to acquire the token.

Outcomes: If the participant flips the *car*-related card (say), they receive the *car* token with a specific (non-zero) probability and the *hat* and the *cat* tokens with zero probability. If the participant receives the *car* token, one of the empty *car* slots is filled and the participant proceeds to the next round of token collection. If a suit's target is reached (say 7 *token* of one type are collected), the participant will receive 10 points for finishing the suit and all of those 7 slots are emptied for a fresh bout of collection.

Game blocks: Gameplay is continuous, and participants retain any tokens from incomplete suits across the entire game. However, the game is structured into blocks, and participants are informed that each block introduces a new probability distribution over the three suits. For example, in an 80-20 block, the probabilities might be 0.8 for *cat*, and 0.2 each for *hat* and *car*. The conditional probabilities of receiving a token given a card-action pairing in such a block are shown in Table 1 (see also Figure 1D). When a new block begins, the suit probabilities shift. For instance, in a 70-30 block, the dominant suit might now be hat, such that $\Pr(hat|\text{hat-related card}) = 0.7$, while $\Pr(cat|\text{cat-related card}) = 0.3$ and $\Pr(car|\text{car-related card}) = 0.3$ (see Figure 1E). Participants are explicitly informed about the block structure: each block lasts 30 rounds, different suits have different probabilities within a block, and these probabilities change when a new block begins.

80-20 block

$\Pr(token\|card)$	***cat***	***hat***	***car***
cat-related card	0.8	0	0
hat-related card	0	0.2	0
car-related card	0	0	0.2

Table 1: $\Pr(token|card)$ for the 80-20 block. The dominant token is *cat* with $\Pr(cat|\text{cat-related card}) = 0.8$

Experimental manipulations

There is a dominant suit in each block. We designed blocks in the game so that there is a dominant suit on each block (counterbalanced across all suit types), such that flipping its cards gives tokens with the highest probability (in Figure 1D, the *cat* suit is the dominant one with 0.8 probability). The

other two suits have identically inferior token probabilities (both *hat* and *car* with 0.2 probability). We instructed the participants of this task manipulation, explaining that within each block there is a specific suit which has the highest chance of token collection. Pursuing the dominant suit type in each block gives participants the best chance at success in suit completion and we investigated how often participants chose the dominant option in a block in relation to their progress on various suits.

Probability disparity between suits is modulated across blocks. We also varied the probability disparity between the dominant and the inferior suits to ascertain how it modulates goal selection. We expected that selection of the dominant suit would be high in the high disparity blocks (where the probability difference between the dominant and the inferior suits is highest) and that this would be reflected in participants' goal selection patterns.

The dominant suit is switched across adjacent blocks. We implemented dominant token switches across blocks so as to enable us to investigate conflicts in goal selection between retrospective and prospective influences, as this is critical to assess how individuals change their choices of goal in the face of such a conflict. If a participant pursues a dominant suit in a block and is close to finishing it, and they encounter a block shift when the hitherto dominant suit is no longer dominant, then it is relatively more difficult to make further progress towards its target. A goal conflict arises in these situations, concerning whether to persist in the suit that is close to the target, or to switch over to the new dominant suit. Such scenarios occur frequently during the game, and the optimal strategy is to switch over to the dominant suit in the new block. We did not instruct the participants that the dominant token in one block becomes an inferior token in the adjacent block.

Testing the effect of instructions. We varied the instructions the participants received on the precise conditional probabilites across blocks. We investigated the extent to which knowledge of absolute probabilities of dominant and inferior tokens (without disclosing the identity of the dominant token) would influence goal selection in the game.

Effect of non-uniform targets on goal selection. We set non-uniform targets for the three suits to ascertain how that influences extended-valuation of the suits in relation to their progress and task-conditions.

We include four experiments to implement the above mentioned design manipulations. Experiments 1-3 have uniform targets for all suits, with each suit comprising of seven tokens of one kind. Experiment 1 has 18 blocks with 30 rounds each, with three different probability structures: 80-20, 70-30, 60-40. In the 80-20 block (say), the dominant suit is available with 80% chance and the two inferior tokens are available with 20% chance. Experiment 2 has 12 blocks (30 rounds each), with two different block types: 75-25 and 55-45. In both experiments 1 and 2, our instructions are limited in that, within each block, one specific suit has the highest probability, and the probabilities change with each block switch. In experiment 3 (which is a repeat of experiment 1), we gave participants more information about their current block context. Before each block began, we specifically informed them that they are in an 80-20 context, without telling them which specific suit is available with 80% chance (more details on task design and instructions are elaborated in the Methods section). In experiment 4, we employed non-uniform targets for different suits and explored its impact on validating our computational hypothesis.

Reviewer 2

I am pleased that the authors addressed most of the reviewers' points in their new submission, which I find much improved compared to the original one. The current manuscript provides a

clearer description of the methodologies, more adequately integrates existing research, and controls for alternative explanations thanks to newly added analyses. However, a couple of points remain to be addressed.

One point that remains unclear is regarding the fitted models' likelihood with respect to participants' goals. In particular, the authors affirm that in rounds where participants do not explicitly declare goals the model selects goals by itself based on their relative values. Such goals are then used to estimate the likelihood of participants' chosen actions, which are known. However, the participants' goals are latent variables, meaning standard model fitting methods based on likelihood may be inappropriate unless integrating over the latent variables across all trials (see e.g., Rmus et al., 2024 Plos Comp Bio). The authors should thus resort to alternative, likelihood-free model fitting methods, or explain why their approach works in the context of their task.

We thank the reviewer for raising this point, and after consideration, we agree with the reviewer's concern. We found the reference on alternative approaches to model fitting using neural networks an interesting read. However, we believe that the reviewer's alternative suggestion of integrating over latent variables across all trials would be the most straightforward way to dispense with the concerns over latent variables not being probed in every round in our paradigm. To address this concern, we implemented a new likelihood-fitting process and thoroughly updated the manuscript to reflect this new method.

Here is a description of the revised likelihood fitting process (now added to the section Model fitting procedure in Methods):

The likelihood of a goal probe is estimated only in the rounds where goal probes are presented in the task (i.e., every third round), whereas the likelihood of an action is estimated in every round. In rounds where the participant does not report their goal, the goal remains latent, and the model estimates selection probabilities across all possible goals. Goal and action likelihoods are then computed by integrating over these latent goal probabilities. The full likelihood estimation process for goal and action selections in the task is summarized as follows.

1. Estimating goal likelihood:

   (a) If the participant's goal is probed in round k (say the goal is *cat*)

   $$\Pr(Goal_k = cat) = 1; \Pr(Goal_k = hat) = 0; \Pr(Goal_k = car) = 0; \tag{1}$$

   (b) For round $k+1$, the latent goal probabilities are estimated given latent goal in k:

   $$\Pr(Goal_{k+1} = G) = \Pr(Goal_{k+1} = G | Goal_k = cat) \tag{2}$$

   Conditional probability of choosing a goal g given the previous goal G is defined as:

   $$Pr(Goal_t = g | Goal_{t-1} = G) = \begin{cases} p_{stay}, & g = G \quad \text{(stay)} \\ (1 - p_{stay}) * softmax(Q_{alt_1}, Q_{alt_2})[g], & g \neq G \quad \text{(switch)} \end{cases}$$

   where p_{stay} is estimated as described in Equation (20), and the softmax is computed over the two alternative goal values.

(c) For round $k+2$, the latent goal probabilities are estimated given latent goal probabilities in $k+1$:

$$\Pr(Goal_{k+2} = G) = \sum_{g} \Pr(Goal_{k+2} = G | Goal_{k+1} = g) * \Pr(Goal_{k+1} = g) \quad (3)$$

(d) The goal is then probed again in round $k+3$, with the goal selection probabilities computed given the latent goal probabilities in $k+2$. This estimation is used to compute the likelihood of goal selection for the participant's goal probe in round $k+3$.

$$\Pr(Goal_{k+3} = G) = \sum_{g} \Pr(Goal_{k+3} = G | Goal_{k+2} = g) * \Pr(Goal_{k+2} = g) \quad (4)$$

(e) The goal probabilities in round $k+3$ are aligned with the latest goal probe (as in step (a)).

2. Estimating action selection probabilities:

$$\Pr(Action_t = A) = \sum_{g} \Pr(Action_t = A | Goal_t = g) * \Pr(Goal_t = g) \quad (5)$$

In this revised model fitting procedure, goal and action likelihoods are estimated since goals are latent in the rounds where they are not probed. In this process, a goal-incongruent action and a switching-of-goal result in different action likelihoods. One limitation of assigning the goal probe reported by the participant to be the true state of the latent goal variable is that goal reports might sometimes be noisy or error prone in disengaged participants. However, the goal probe statistics we report assuage this concern because of the high congruence found between self-reported goals and goal-consistent actions observed in participants' data (see Supplementary Fig. S2).

We revised the parameter and model recovery figures using this new procedure. In the recovery process, the simulated model reports a goal every third round similar to the participants. We also updated all the figures where model-fitted parameters were used to generate the data used in the figures. The revised figures are captioned in red in the highlighted manuscript. We also added a revised description of the new procedure in the Methods section. Note that our conclusions in the manuscript do not change significantly compared to that presented in the previous version of the manuscript. Some of the specific statistical results were updated wherever necessary.

We thank the reviewer for their suggestion about the model fitting process, which makes the fitting procedure more theoretically robust.

Moreover, is it correct that the model (algorithm 1) decides whether to switch goals based on the values of the current and the best alternative goal, but that in the case of a switch, the model could select the third available goal? If so, could the authors please justify this choice?

Yes, the model makes the decision to stay with a probability (p_{stay}) based on the difference between the current goal value and the best alternate goal value.

When it decides to switch away from the current goal with the probability $(1 - p_{stay})$, it selects a new goal from the two alternate goals depending on the values of those two alternate goals ($softmax(Q_{alt_1}, Q_{alt_2})$)

The new selection probability of an alternate goal 1 in case of a switch would therefore be:

$$\Pr(Goal = Q_{alt_1}) = (1 - p_{stay}) * softmax(Q_{alt_1}, Q_{alt_2})[alt_1] \quad (6)$$

This ensures exploration of the third goal even though stay/switch decisions were based on the best of the two alternate goals. This still highly values the best alternate goal if the value difference between two alternate goals is high. We amended Algorithm 1 in the manuscript to include this detail.

We opted to use the stay/switch mode of decision-making as a way to frame the modeling process, given that a similar approach is used routinely in the foraging literature [1]. We considered that the relatively stable nature of an individual's commitment to a particular option (patch) in a foraging task resembles the relatively stable commitment to a goal we observed in the current paradigm. Thus, we considered it useful to adopt a similar modeling framework (stay/switch) in the present study.

I also have some minor suggestions.

In some figures with model simulations, e.g., 2 and 4, the authors add the "optimal" specification to prospective and retrospective models to specify these models were not fit on participants' behavior. However, I believe optimized would be a more appropriate term, especially for the retrospective model, since it is not optimal for task performance.

We replaced 'optimal' with 'task-optimized' in these figures.

In addition, could the authors please state somewhere how many iterations per real participant were run by the models in order to produce simulated data?

One sample of synthetic data per real participant is generated using the fitted parameters and the aggregate choice patterns of the population of model-simulated participants are contrasted with that of the real participants.

We added this in the section, *The TD-momentum model reproduces behavioral effects of the aggregate participant pool.*

On page 14, the authors state the prospective agent shows a "clear" interaction of performance with block type. However, I think "significant" would be a more appropriate adjective.

This is corrected.

In general, I think the authors would benefit from additional proofreading and editing, as I noticed several typos/inconsistencies that should be corrected before the final submission.

We thank the reviewer for pointing this out. We have carefully proofread the manuscript and corrected all identified typos and inconsistencies to improve clarity and readability.

References

[1] Sara M. Constantino and Nathaniel D. Daw. Learning the opportunity cost of time in a patch-foraging task. *Cognitive, affective & behavioral neuroscience*, 15:837–853, 12 2015.
